# Amorphous Ribbon Brazing Filler Metals: Selection Criteria, Glass Formation, and Transient Liquid Phase Bonding

**DOI:** 10.3390/ma19194123

**Published:** 2026-09-26

**Authors:** Alexander Ivannikov, Sofya Terekhova, Alexey Suchkov, Ivan Fedotov, Nikita Popov, Ivan Klyushin, Pavel Morokhov, Oleg Sevryukov

**Affiliations:** 1 Department of Materials Science, National Research Nuclear University MEPhI (Moscow Engineering Physics Institute), Kashirskoe Shosse 31, 115409 Moscow, Russia; aaivannikov@mephi.ru (A.I.); nspopov@mephi.ru (N.P.); i348hob2@yandex.ru (I.K.); ivannikov7@rambler.ru (P.M.);; 2 MIFI-AMETO LLC., Kashirskoe Shosse 31, 115409 Moscow, Russia

**Keywords:** amorphous ribbon brazing filler metal, rapidly quenched ribbon, glass-forming ability (GFA), transient liquid phase (TLP) bonding, eutectic, melt spinning, active brazing, titanium, zirconium, nickel, copper–phosphorus filler metals

## Abstract

Amorphous ribbon brazing filler metals produced by rapid melt quenching form a distinct class of high-temperature brazing materials; a few ductile microcrystalline ribbons are treated as boundary cases. This review systematizes the four industrial families of such fillers: copper–phosphorus (Cu–Ni–Sn–P), titanium (Ti–Cu–Ni, Ti–Zr–Cu–Ni(–Be)), zirconium (Zr–Ti–Cu–Ni, Zr–Cu–Fe–Be), and nickel–metalloid (Ni–Cr–Si–B, Ni–Cr–P) alloys, together with the closely allied iron-based fillers. This selection is no accident: to become the basis of a commercial filler metal, an amorphous alloy must simultaneously satisfy five criteria (C1–C5) of brazing temperature, glass formation, base-material compatibility, ribbon manufacturability, and cost and safety, which are formulated here as a single cross-system checklist with operational thresholds. The class itself exists because in deep eutectics, the depressant and glass-former functions can coincide. Transient liquid phase (TLP) bonding, for which the thin homogeneous ribbon is a particularly suitable carrier, runs through the review as a unifying thread. The structural heredity of the ribbon-derived melt and the complementarity of the families by base material are discussed; ductile silver and aluminum fillers serve as a negative control delineating the boundary of the class, and the criteria are applied predictively to compare the families and to select a filler metal for a specific task.

## 1. Introduction

High-temperature brazing remains one of the principal methods for the permanent joining of materials in modern engineering and complements welding where the latter is fundamentally inapplicable. High-temperature brazing is understood here as joining with filler metals whose liquidus temperature exceeds 450 °C, whereas soldering, for example with tin-based solders (temperatures below 450 °C, covered by the separate standard ISO 9453 [1]), is outside the scope of the present review. The applications in question include joining dissimilar metal pairs, joining metals to ceramics and graphite, producing large numbers of joints in a single heating cycle, and structures for which localized welding heat is unacceptable [2,3,4,5]. Industrially, the automotive industry (heat-exchange equipment) is the dominant consumer of brazing filler metals, while the fastest growth is attributed to the aerospace and power-engineering sectors [6,7]. The requirements imposed on filler metals are tightening in all directions at once: service temperatures and corrosion resistance of the joints, quality reproducibility in series production, and compliance with environmental restrictions on toxic components are all subject to ever stricter demands. Under the REACH regulation (Registration, Evaluation, Authorization and Restriction of Chemicals), cadmium in brazing filler metals has been restricted in the European Union since 2011 [8,9]. This has effectively removed cadmium-bearing silver filler metals from circulation. The toxicity of beryllium and its compounds [10,11] constrains the use of beryllium-containing compositions. The classification of nickel compounds as carcinogenic to humans [11] is one of the drivers behind the iron-based filler metals that are meant to replace nickel-based ones [12,13,14].

The current nomenclature of high-temperature brazing filler metals is codified in the AWS A5.8M/A5.8 [15] standard and in the parallel ISO 17672 [16], which together cover more than 120 compositions grouped by base. Silver-based BAg filler metals have historically served as the universal solution for steels, copper, and cemented carbides at moderate brazing temperatures. Copper BCu and copper–phosphorus BCuP grades were created for the furnace brazing of steels and the self-fluxing brazing of copper in heat-exchange engineering. Gold-based BAu and palladium-based BPd grades were developed for aerospace and vacuum assemblies with stringent requirements for ductility and seam purity. Nickel-based BNi filler metals provide heat-resistant, corrosion-resistant joints in steels and nickel superalloys; titanium BTi grades are intended for titanium structures and active brazing; aluminum BAlSi grades serve aluminum heat exchangers; and cobalt BCo and magnesium BMg grades cover the narrow niches of heat-resistant and lightweight structures [4,17].

A substantial share of the most widely used filler-metal compositions are eutectic alloys whose room-temperature structure contains a considerable fraction of brittle phases. Among them are copper-, nickel-, and iron-based alloys with metalloid depressants (phosphorus in the copper alloys; boron, silicon, and phosphorus in the nickel and iron alloys), whose structure contains phosphides, borides, and silicides [5,17,18]. Also of major industrial importance are active filler metals based on titanium and zirconium with additions of copper, nickel, and beryllium, in which the brittleness stems from intermetallics (Ti_2_Cu, Ti_2_Ni, Zr_2_Cu, including complex ones such as (Ti,Zr)_2_(Cu,Ni)) [19,20,21]. In the as-cast state, none of these alloys can be rolled or drawn, and for decades they were used as powders, pastes, and cast preforms, which limited the accuracy of filler-metal dosing, required organic binders, and degraded joint quality [5,22,23]. The situation was changed by the technology of rapid quenching from the melt. Melt spinning onto a rotating wheel at cooling rates of 10^5^–10^6^ K/s makes it possible to produce, from these brittle eutectic alloys, a continuous ductile (flexible) ribbon, typically 18–60 μm thick, in the amorphous or nanocrystalline state [24,25] (the nanocrystalline case is confined to a few ductile compositions and lies outside the formal scope defined in Section Scope and Literature Search Methodology). The principle of the process is shown in Figure 1.

The first commercial amorphous ribbon brazing filler metals, the MBF (Metglas Brazing Foil) series, were developed by Allied Signal in the late 1970s [5,19]. In Russia, a similar line of work has been pursued since 1992 at the National Research Nuclear University MEPhI and has been implemented in the series-produced STEMET grades [26,27,28]. Today, amorphous ribbon filler metals are series-produced by Metglas (Conway, SC, USA), Vacuumschmelze (Hanau, Germany, VITROBRAZE series), VBC Group (Loughborough, UK), and AT&M (Beijing, China); in Russia, they are produced by MIFI-AMETO LLC (Moscow, Russia; STEMET) and GAMMAMET (SPAYMET). The product ranges of the series and their manufacturers are surveyed in Section 5.7 and Section 7.6.

The advantages of the amorphous ribbon form over powders and pastes are systemic. First among them is chemical and structural homogeneity: the amorphous ribbon is free of macrosegregation and of any binder, and its content of non-metallic inclusions is far lower than that of atomized powders; it melts within a narrow range and provides uniform wetting over the entire joint area. The second advantage is dosing accuracy, since the ribbon geometrically defines the brazing clearance, while its small thickness minimizes the amount of melt and thereby the erosion of the base metal and the volume of brittle phases in the seam. The third advantage is manufacturability, as preforms of complex shape can be stamped from the ductile ribbon, which is critical for the series assembly of heat exchangers, honeycomb panels, and engine components. Finally, the ribbon form is particularly well suited to transient liquid phase (TLP) bonding. Under this brazing regime, the limited reserve of melting-point depressants in a thin ribbon has time to diffuse into the base material within technologically acceptable holding times, and the seam acquires a solid-solution structure with a remelt temperature above the brazing temperature [29,30,31,32]. These aspects are considered in detail in the subsequent sections of the review.

It is important to emphasize that amorphization is not an end in itself but a forced, and at the same time exceptionally successful, technological solution for a specific class of alloys. Ductile filler-metal systems such as Ag–Cu (with additions of Zn, Cd, Sn, In, and others), Au–Ni (Pd, Ag, and others), and Al–Si (Cu, Mg, Mn, and others) can, for the most part, be rolled into foil by conventional methods [33,34]; rapid quenching offers them no decisive advantages, while their base systems possess low glass-forming ability. These systems, which delineate the applicability boundary of the amorphous form, are considered in Section 4. The true niche of amorphous ribbons remains the brittle eutectic systems in which the elements responsible for low melting points are simultaneously glass formers. As shown in Section 2, this coincidence is not accidental and defines the entire character of this class of materials.

A number of review papers have been devoted to amorphous filler metals, but their coverage is fragmentary. The classic reviews by Rabinkin [5,18] summarized the experience of the first decades of MBF ribbon use but do not reflect the results of the last twenty years. Modern reviews either cover all filler metals without emphasis on the product form [17] or are confined to a single family, be it nickel-based filler metals for austenitic steels [32], titanium filler metals [20], or the joining of zirconium alloys [21]. A cross-system analysis of amorphous ribbon brazing filler metals as a unified class of materials, with common composition criteria, a single “glass formation–brazeability” logic, and a comparison of the families by application, is absent from the literature. The present review is intended to fill this gap. Amorphous ribbon filler metals are not distinguished as a class even in the industry classifications AWS A5.8/ISO 17672 [15,16] (Section 8), so their description requires an analytical framework of its own.

The contribution of the present review relative to these surveys is therefore not a new set of physical rules but their explicit organization. Criteria C1–C5 (Section 2.2) synthesize selection rules that are scattered across the literature on nickel [5,18], titanium [19,20], and zirconium [21] filler metals and across the metallic-glass literature [35,36,37]. To our knowledge, they have not previously been formulated as a single checklist and applied at the same time to all four industrial families and to the systems that failed the selection. Three elements of the framework are, we believe, new. The first is the separation of glass-forming ability (C2) from the industrial processability of the ribbon (C4), which the Ni–Si–Be and zirconium-based ribbons show to be independent (Section 2.2, Section 4.3 and Section 7.4). The second is the use of ductile silver and aluminum fillers and of record glass formers (Vitreloy, Pd–Ni–P) as negative controls (Section 2.3 and Section 4). The third is a cross-family comparison of the kinetics of transient liquid phase bonding (Section 5.4, Section 6.1, Section 7.5 and Section 8). A further element is the systematic inclusion of the results of the Russian school of rapidly quenched filler metals (the STEMET grades and the associated dissertations and journal papers). Most of them have not previously been brought together in the English-language literature. These data are marked as such and are presented alongside the data on their foreign counterparts.

The review is organized as follows. Section 2 formulates criteria C1–C5, applies them to the known amorphous families, and sets out the TLP mechanism common to the metallic base materials (Section 2.4), separating the systems that became industrial ribbons from the borderline and screened-out ones. Section 3 covers the copper–phosphorus ribbons (Cu–Ni–Sn–P), and Section 4 uses the aluminum and silver systems to delineate the applicability boundary of the amorphous form. Section 5, Section 6 and Section 7 consider the titanium, zirconium, and nickel and iron families, including TLP bonding and the use of each family beyond its native base materials. Section 8 brings the families together in a single comparative field.

### Scope and Literature Search Methodology

This review covers brazing filler metals that are produced industrially, or have been demonstrated at the laboratory scale, as rapidly quenched ribbons whose as-cast state is amorphous, that is, X-ray amorphous or amorphous with a minor nanocrystalline fraction, and whose liquidus temperature exceeds 450 °C. Ductile ribbons obtained by rapid quenching in a nanocrystalline or microcrystalline state (Ni–Si–Be, Al–Ge–Si, Al–Si) and conventionally rolled foils (Ag–Cu–Zn, Ag–Cu–Ti, Ni–Mn) lie outside this formal scope; they are discussed only as comparison cases that delineate the boundary of the class and are identified as such wherever they appear. The review is narrative rather than systematic in the PRISMA sense, but the literature was collected according to the structured procedure described below.

The core of the reference collection was assembled by the authors’ group over the period 1992–2026 and is maintained in a Mendeley library. It was compiled by manual searches in Scopus, Web of Science, eLibrary.ru, and Google Scholar, in English and Russian. It includes the monographs, handbooks, dissertations, and journal papers of the Russian brazing school that are not indexed in the international databases. For the present review this collection was supplemented in 2026 by a systematic sweep of the period 1960–2026, with emphasis on 2000–2026 and a targeted update for 2024–2026. The sweep used the Crossref, OpenAlex, and Semantic Scholar databases with forward and backward citation chaining from the reviews of Rabinkin [5,18], Way et al. [17], Li et al. [20], and Jiang et al. [21]. It also covered the publisher platforms ScienceDirect, SpringerLink, Wiley, Taylor & Francis, and MDPI, the patent databases Google Patents, Espacenet, and USPTO, the current editions of the AWS A5.8M/A5.8 [15] and ISO 17672 [16] standards, and the product catalogs and safety data sheets of the ribbon manufacturers (Metglas, Vacuumschmelze, VBC Group, Titanium Brazing, MIFI-AMETO, and the Ashinskiy Metallurgical Works). The search terms combined product-form descriptors (“amorphous brazing foil”, “amorphous brazing ribbon”, “rapidly quenched filler metal”, “melt-spun filler metal”, “metallic glass filler”) with family descriptors (Cu–Ni–Sn–P, Ti–Cu–Ni, Ti–Zr–Cu–Ni, Zr–Cu–Be, Ni–Cr–Si–B, Ni–Cr–P, Fe–Cr–Si–B, Fe–Cr–P) and with process descriptors (“transient liquid phase”, “isothermal solidification”, “diffusion brazing”, “active brazing”, “glass-forming ability”), together with their Russian equivalents.

A source was included if it (i) reported the composition and melting range of the filler metal, (ii) specified the product form (ribbon, foil, powder, or paste), and (iii) reported at least one of the following: glass-forming characteristics, seam microstructure, joint properties, or an industrial application. Studies of bulk metallic glasses without a brazing context were included only where they establish the glass-forming ability of a system relevant to a filler-metal family or serve as a negative control, and work on soldering below 450 °C was excluded. Manufacturers’ data are cited as such and are distinguished in the tables from peer-reviewed data. The data on the STEMET grades obtained by the authors’ group are cited to the publications in which they were reported; no unpublished data are presented.

## 2. Glass Formation and Brazeability: Selection Criteria for Amorphous Systems as Brazing Filler Metals

In the six decades since the first metallic glasses were obtained by quenching from the melt [24], the amorphous state has been realized experimentally in hundreds of metallic systems, from classical metal–metalloid alloys to bulk metallic glasses (BMGs) with critical diameters of tens of millimeters [35,38]. Against this background it is remarkable that only a few families of amorphous alloys have become established as industrial brazing filler metals, namely the copper–phosphorus (Cu–Ni–Sn–P), titanium (Ti–Cu–Ni, Ti–Zr–Cu–Ni), zirconium (Zr–Ti–Cu–Ni, Zr–Cu–Fe–Be), and nickel/iron–metalloid (Ni–Cr–Si–B, Ni–Cr–P, Fe–Cr–Si–B, Fe–Cr–P) families. The aim of the present section is to show that this selection is not accidental and to formulate a system of criteria that an amorphous system must satisfy simultaneously in order to serve as the basis of a ribbon filler metal. These criteria are then used as the analytical framework of the entire review.

### 2.1. Deep Eutectics and the Coincidence of Filler-Metal and Glass Compositions

The design philosophy of a brazing filler metal is built on deep eutectics. The brazing temperature must lie below the degradation temperature of the base material, so melting-point depressants, elements that sharply lower the melting temperature, are introduced into the filler-metal base. In copper systems the depressant role is most often played by P and Sn; in titanium and zirconium systems it is performed by Cu, Ni, and occasionally Be; in nickel systems, by the metalloids B, P, and Si. The filler-metal compositions optimal in terms of low melting point lie near the eutectic points, and it is also near deep eutectics that the glass-forming ability of most filler-metal systems reaches its maximum, although the two extrema do not coincide exactly (see below). Thus the composition of a good filler metal and the composition of an alloy prone to amorphization coincide in many cases and correspond to the same region of the phase diagram. The coincidence, however, is asymmetric. A deep eutectic is a strongly favorable, but not a sufficient, condition for glass formation. It is shown below that both properties feed from the same thermodynamic root, yet the glass requires additional conditions that the eutectic does not.

Both the type of phase diagram and the ability to vitrify are governed by the character of the physicochemical interaction between the components. Formally, this is reflected in how the free-energy curve of the liquid G_L_(x, T) is positioned relative to the curves of the crystalline phases G_S_(x, T). The shape of these curves is set by the mixing energy ω (the interchange energy), expressed through the pairwise interatomic interaction potentials:

ω = ε_AB_ − 0.5·(ε_AA_ + ε_BB_) (1)

where ε_AB_, ε_AA_, and ε_BB_ denote the bond energies of unlike and like atomic pairs. The sign of ω determines the character of the system and the type of phase diagram. At ω ≈ 0, when the sizes and structures of the components are similar, an ideal solution with unlimited mutual solubility forms (Cu–Ni); there is no eutectic, and the tendency toward vitrification is minimal. At ω > 0 the unlike bonds are weaker than the like ones, and the system tends toward clustering and demixing, which yields limited solid solubility and a simple eutectic (Ag–Cu). At ω < 0 the unlike bonds are stronger, and the system tends toward ordering and the formation of intermediate phases. Importantly, eutectics arise for both signs of nonzero ω. At ω > 0 the eutectic is simple and shallow, whereas at ω < 0 it is deep and lies adjacent to intermediate compounds (Figure 2).

The eutectic point corresponds to the location on the phase diagram where the liquid is maximally competitive in free energy with respect to all available crystalline phases. The driving force for crystallization is minimal there, and the liquidus line is depressed to its lowest point. This provides a direct bridge to Turnbull’s classical glass-formation criterion, which relates the tendency of a melt toward amorphization to the reduced glass transition temperature T_rg_, equal to the ratio of T_g_ to T_liq_. The higher T_rg_ (roughly T_rg_ ≥ 2/3), the narrower the temperature interval in which the melt must bypass crystallization, and the lower the required cooling rate [36]. Since the glass transition temperature T_g_ depends only weakly on composition within a given system, whereas the liquidus temperature T_liq_ drops sharply near eutectics, the maxima of glass-forming ability (GFA) tend to fall near deep eutectics. This is a tendency rather than a law. In the Cu–Zr system the best binary glass formers, Cu64Zr36 and Cu50Zr50, lie at compound rather than eutectic compositions [39,40], and in Ti–Cu the GFA maximum at Ti50Cu50 does not correspond to the deepest eutectic (Section 5.1); such deviations are attributed to the structural factor discussed below. Note that the regular-solution formulation in terms of ω is a mean-field approximation and is used here qualitatively, as a language for describing the sign of the deviation from ideality, not as a computational model.

The eutectic criterion, however, is a purely equilibrium one: it describes the shape of the phase-diagram lines. Amorphization, by contrast, is governed by kinetics. The outcome of quenching depends on the cooling rate and on whether crystals have time to nucleate and grow during solidification, so the melt must supercool faster than these processes can develop. The difference is already visible in the sign of ω. A eutectic formed at a positive deviation from ideality (ω > 0, a tendency toward demixing in the melt) remains simple. Its crystallization kinetics are fast, and virtually no glassy phase forms on quenching (Ag–Cu). The best glass former turns out to be a eutectic with a negative enthalpy of mixing in the melt (ω < 0, strong chemical short-range order) [41] combined with limited solid solubility due to atomic size mismatch.

The reason lies in so-called frustration, that is, the incompatibility of the melt’s preferred local order with the available crystal lattices. The melt strives toward a local configuration with strong A–B short-range order that no crystal lattice available at the given composition can realize without a change in stoichiometry or symmetry; the transformation into a crystal therefore requires destroying this short-range order and overcoming a high nucleation barrier, and the melt kinetically freezes into a glass. This is precisely why the best glass formers combine a large atomic size mismatch with a negative enthalpy of mixing. The mismatch simultaneously limits solid solubility, deepening the eutectic, and destabilizes the crystal lattice, favoring the glass [35,37,42,43].

The tendency of an alloy toward amorphization is described quantitatively by a set of reduced temperatures [44].

The reduced melting temperature:

t_m_ = k·T_m_/ΔH_evap_ (2)

Here ΔH_evap_ denotes the heat of evaporation, which serves as a measure of the interatomic bond energy, and k the Boltzmann constant (the heat of evaporation is taken per atom, so that t_m_ is dimensionless). The smaller tm, that is, the stronger the bonding relative to the thermal energy at melting, the higher the melt viscosity at the liquidus and the higher the glass-forming ability of the alloy. This thermochemical estimate is consistent with the Turnbull criterion and does not replace it; both measures point to the same deep eutectics.

The reduced supercooling:

ΔT_mr_ = (T_mI_ − T_m_)/T_mI_, where T_mI_ = 0.5·(T_mA_ + T_mB_) (3)

Increases as the melting temperature of the alloy itself decreases and as the melting temperatures of its pure components increase. Here T_m_ denotes the melting temperature of the alloy, T_mA_ and T_mB_ the melting temperatures of its pure components, and T_mI_ their mean value. ΔT_mr_ is therefore maximal precisely for deep eutectics of high-melting metals. Structural and electronic factors add to this. Stable glass formation requires an appreciable atomic size mismatch and favorable differences in the valences and electronegativities of the components, and what matters is falling within an optimal range rather than maximizing each factor separately [35,37]. For metal–metal systems of the Ti–Cu and Zr–Cu type, rigid empirical numerical windows for these parameters are not strictly obeyed, so generalized criteria that account simultaneously for packing topology and chemical interaction are more reliable.

Modern practice supplements the Turnbull criterion with computational metrics of glass-forming ability. The width of the supercooled liquid region ΔT_x_ equals the difference between the crystallization onset and glass transition temperatures, and a second widespread measure is the parameter

γ = T_x_/(T_g_ + T_liq_) (4)

where T_g_ denotes the glass transition temperature, T_x_ the crystallization onset temperature on heating, and T_liq_ the liquidus temperature. Both parameters correlate well with the critical cooling rate across hundreds of alloys [45], and in recent years the screening of glass-forming compositions has been accelerated by machine learning methods that combine such parameters with high-throughput experiments [46]. These criteria are of four different kinds and are complementary rather than equivalent: the thermodynamic criterion of Turnbull and the reduced temperatures of Equations (2) and (3) describe the driving force for crystallization, the structural criteria (atomic size mismatch, cluster packing [43]) describe how difficult it is to build a crystal from the local order of the melt, the kinetic factor decides whether nucleation has time to occur at the actual cooling rate, and ΔT_x_ and γ are empirical measures of the observed outcome. For brazing purposes what matters is that, with the reservations noted above, all four point to the same region of deep eutectics. In the language of the criteria of Section 2.2, the depth of the eutectic is what satisfies C1, and the glass-forming factors listed here are what decide C2; the remainder of this section shows that the two can, but need not, be satisfied by the same composition.

Where do the industrial ribbons stand on this scale? Table 1 answers this question for the three eutectic systems compared below and for the copper–phosphorus system of Section 3. For the ribbons the glass transition is not resolved. At the heating rates of 10–30 K/min used in routine DTA and DSC the crystallization exotherm appears before any glass-transition endotherm, so ΔT_x_ and γ cannot be assigned to them. In practice their glass-forming ability is described by the crystallization temperature T_x_ and by the critical ribbon thickness t_cr_, and this is why criterion C2 in Section 2.2 is formulated through t_cr_ rather than through γ. The comparison with the bulk glasses is nevertheless instructive. Vitreloy 1 and Pd40Ni10Cu30P20 have a supercooled liquid region of 80–95 K and γ of 0.44–0.48, and they can be cast as rods 10–40 mm in diameter. The nickel and copper–phosphorus ribbons crystallize at 250–500 °C, several hundred degrees below their solidus, and remain amorphous only up to 50–70 μm. Even the best-studied Ni–Si–B glasses show a distinct glass transition only after alloying with 4 at.% Nb or Ta, and then with ΔT_x_ of 30–35 K and T_g_/T_liq_ of 0.59–0.61 [47]. The class of amorphous filler metals is thus made up of marginal glass formers. What defines it is not a high glass-forming ability but the coincidence of the depressant and glass-former functions.

Yet even the concordance of all the reduced temperatures does not guarantee amorphization, because kinetics has the final say. The following example is instructive. The interaction of Fe with Si is stronger than that of Fe with B, and by the criterion of interatomic bond strength the Fe–Si system should amorphize more easily. In practice the opposite is true. binary Fe–Si melts have not been amorphized by melt quenching at any composition, whereas Fe–B amorphizes readily over the range of about 12–28 at.% B [44,48,49]. Two reasons can be suggested, and both belong to the domain of kinetics and topology. First, in the association model of liquid alloys [50] the decisive role is played not by the intensity of the interaction as such but by the entropy of formation of associative complexes in the melt; a more negative entropy of formation of the Fe_3_B-type complexes than of the Fe–Si complexes would explain the different crystallization kinetics on quenching. We present this as an interpretation, since the two systems have not been compared quantitatively in these terms. Second, the diagram topologies differ [51]. In Fe–B a deep eutectic (near 17 at.% B) is combined with virtually zero solubility of boron in α-Fe; the crystal simply has nowhere to accommodate the boron. In Fe–Si the liquidus is shallow, the solubility of silicon is high, and the B2- and D03-ordered superstructures of the body-centered cubic (bcc) solution (up to Fe_3_Si) provide an easy crystallization path. The Fe–B system reproduces the glass-favorable case of “ω < 0 with limited solid solubility.” In the Fe–Si system the crystalline solid solution readily accepts the second component, so on quenching crystallization outruns vitrification. A comparison of four characteristic systems, separating the axes “deep eutectic,” “amorphizability,” and “suitability as a filler metal,” is compiled in Table 1. Along these axes the four systems read as follows. Ag–Cu has a eutectic but no glass (ω > 0). Cu–Ni–Sn–P and Ni–Si–B combine the eutectic and the glass (ω < 0 with a large size mismatch), the copper–phosphorus system being the more marginal glass former of the two. Zr–Cu(–Be) has the glass, but its use in brazing is limited by factors outside the structure.

**Table 1:** Comparison of four eutectic systems in terms of thermodynamics, glass-forming ability, and status in brazing.

Attribute	Ag–Cu(–Sn)	Cu–Ni–Sn–P	Ni–Si–B	Zr–Cu(–Be)
Depressant	Cu, Sn	P (Sn, Ni)	B, Si, (P)	Cu, (Be)
Sign of ω (enthalpy of mixing in the melt)	ω > 0 (tendency toward demixing in the liquid, eutectic without compounds)	ω < 0 (Cu–P: deep eutectic at 714 °C next to Cu_3_P)	ω < 0 (strong attraction between unlike atoms, deep eutectic with borides and silicides)	ω < 0 (strong attraction between unlike atoms, eutectics with intermetallics)
Type of eutectic	simple, with limited solid solubility	metal–metalloid, with phosphides (Cu_3_P, Ni_3_P, Ni_2_CuP)	metal–metalloid, with borides and silicides	with intermetallics (Zr_2_Cu and others)
Atomic size mismatch	moderate (Ag/Cu ≈ 12%)	moderate (P is smaller than Cu by about 15%)	large (the B atom is smaller than Ni by about 25%, and Si by about 10%)	large (Zr is larger than Cu by about 25%, and Be smaller than Zr by about 30%)
Glass-forming ability (critical thickness of the fully amorphous product)	low: ribbon does not amorphize on melt spinning	moderate: amorphous ribbon 50–60 μm (STEMET 1101); binary Cu–P only 20–35 μm [52]	high: amorphous ribbon 60–70 μm (STEMET 1301) [52,53]	record high: rods ≥ 10 mm (Vitreloy 1) [54]; 40 mm (Pd40Ni10Cu30P20) [55]
T_g_, T_x_, and T_liq_ (heating at 10–30 K/min)	no glass forms on melt spinning	T_g_ not resolved; T_x_ = 250 °C (first stage) and 315 °C (second stage), STEMET 1101 [52,56]; T_x_ = 190–270 °C, Cu–Ni–P foils [57]; T_liq_ = 650 °C (STEMET 1101 [53]) and 655 °C (MBF-2005 [58])	T_g_ not resolved in the industrial ribbons; T_x_ (onset) = 491 °C (STEMET 1301), 471 °C (STEMET 1309), and ≈475 °C (STEMET 1342), DSC at 20 K/min [30,32,52]; T_liq_ = 1020 °C (STEMET 1301 [53]) and 1024 °C (BNi-2 [15]); T_g_ resolved in Ni75Si8B17 only after alloying with 4 at.% Nb or Ta [47]	T_g_ = 352 °C, T_x_ = 432 °C, T_liq_ = 720 °C, Vitreloy 1 [54]; T_g_ = 302 °C, T_x_ = 397 °C, T_m_ = 531 °C (as reported), Pd40Ni10Cu30P20 [55]
ΔT_x_ = T_x_ − T_g_ and γ = T_x_/(T_g_ + T_liq_)	—	ΔT_x_ and γ not reported for Cu–Ni–(Sn)–P glasses (T_g_ not resolved); for the nearest studied phosphorus glass, binary Ni80P20, T_g_ = 322 °C, T_x_ = 344 °C, ΔT_x_ = 22 K [59]	ΔT_x_ and γ not defined for the ribbons; ΔT_x_ = 30–35 K, T_g_/T_liq_ = 0.59–0.61, Nb- and Ta-alloyed Ni75Si8B17 glasses [47]	ΔT_x_ = 80 K, γ = 0.44, Vitreloy 1; ΔT_x_ = 95 K, γ ≈ 0.48, Pd40Ni10Cu30P20 [45]
Product form and status as a filler metal	rolled ductile foil; mass-produced (BAg class), amorphization not needed	amorphous ribbon 25–60 μm (melt spinning); industrial (MBF-2005, STEMET 1101), silver-free counterpart of the BCuP class	amorphous ribbon 40–60 μm (melt spinning); industrial (BNi class; MBF, STEMET)	rods up to tens of millimeters in diameter (Vitreloy) and ribbon 20–50 μm; niche, limited by Be toxicity and cost

Note: Ag–Cu serves as the reference eutectic arising from a positive deviation and hence as a mediocre glass former; this system is treated as a negative control in Section 4 (the 72 wt.% Ag eutectic at 779 °C). Ni–Si–B corresponds to the working niche of amorphous ribbons, where the depressant simultaneously acts as the glass former (Section 7). The Zr–Cu–Be family of bulk glasses of the Vitreloy type [54] demonstrates that record glass-forming ability by itself does not make an alloy an industrial filler metal. Sources. The signs of ω and the types of eutectic follow the phase diagrams [51] and the mixing-enthalpy classification of Takeuchi and Inoue [41]; the atomic size mismatches are from the radii tabulated in [42]; the glass-forming ability and product forms are from [5,53,54,60] and Section 7.1; the summary of the four systems along these axes, given in the text before the table, is the authors’ assessment. The ribbon data are measurements of the authors’ group (DTA, argon, 20 K/min [52]; DSC, Netzsch STA 409 CD, 20 K/min, helium, alumina crucibles with lids), supplemented by [56]. The bulk-glass data are from [45,54,55]. Pd40Ni10Cu30P20 contains no zirconium; it is listed in the Zr–Cu(–Be) column only as a second reference bulk glass with record glass-forming ability.

Importantly, the best glass formers almost never become brazing filler metals, and the reasons lie outside glass formation. Bulk Zr–Ti–Cu–Ni–Be glasses of the Vitreloy type hold the record for glass-forming ability and are cast as rods of centimeter diameter [54], yet beryllium bars them from mass-market brazing on toxicity grounds, and their cost leaves them a material for one-off products. Pd–Cu–Ni–P glasses amorphize into ingots up to 40 mm in diameter at cooling rates of the order of a few kelvin per second [55], but a palladium-based filler metal is priced out of series brazing. Rare-earth-containing glasses, from La–Al–Ni to the aluminum-based Al–REM–TM (REM = rare-earth metals, TM = transition metals) glasses of the same group [61], remain laboratory exotica. They are expensive, their chemical compatibility with structural base materials has not been studied, and the rare-earth elements do not perform the depressant function. Ductile Cu–Zr–Al glasses [62] are of interest for particular tasks, but in terms of seam chemistry they adjoin the zirconium family, whose industrial niche is small. The conclusion is the same for all groups. Record glass-forming ability does not substitute for meeting the selection criteria, and the class of industrial ribbons is formed not by the best glass formers but by alloys that combine glass formation with the brazing function. For brazing, the opposite situation matters more: an alloy that needs no record glass-forming ability but does need the ribbon form. A eutectic alloy that is difficult or impossible to roll into foil because of brittle intermetallics, borides, and phosphides can in a number of cases be obtained as a thin ductile ribbon by melt spinning [5]. A further observation concerns the melt itself. We present it as an open question rather than as an established property of the class. The kinematic viscosity of melts obtained from rapidly quenched and from crystalline filler metals of the same composition was measured directly for STEMET 1101 (Cu base, 9–10% Ni, 3–4% Sn, 7–8% P) and STEMET 1301 (Ni base, 7% Cr, 3% Fe, 4.5% Si, 3% B), compositions in wt.%. Immediately after melting, the viscosity of the melt obtained from the amorphous ribbon was 25–50% higher than that of the melt obtained from a crystalline ingot. The difference persisted up to the melt homogenization temperatures of 1000–1170 °C, well above the typical brazing superheat of 30–50 °C [52,63]. A similar dependence of melt viscosity on the initial state of the ribbon has been reported independently for the glass-forming iron-based alloy Fe72.5Cu1Nb2Mo1.5Si14B9 (at.%, a Finemet-type composition) [64]. The initial state also affects the onset of crystallization on cooling: for three Cu–Ni–Sn–P compositions the melts obtained from ribbon and from ingot began to crystallize at temperatures that differed by 20–77 °C (Section 3.3) [56]. These are the only alloys for which such measurements are known to us, so the observation cannot yet be generalized, and its interpretation is open. In the literature on metallic melts, effects of this kind are attributed to a kinetically long-lived microheterogeneity of the melt, that is, to clusters on the nanometer and submicron scale inherited from the initial state and destroyed only by substantial superheating [65,66]. A contribution from inherited oxide inclusions and from the dispersity of the charge cannot be ruled out, and experiments separating these factors have not been performed. We therefore treat the “structural heredity” of the ribbon-derived melt as a hypothesis worth testing rather than as a property of the class.

A related qualitative observation concerns wetting. Laid in a single layer on the base material, a ribbon 40–50 μm thick does not gather into a droplet on melting but spreads over the surface with a contact angle close to zero. This has been noted repeatedly in the production practice of the authors’ group [52,67] but has not been quantified by sessile-drop measurements, and we present it as a qualitative observation rather than as evidence. The geometry of a thin layer, with its large surface-to-volume ratio and a melt film that is already in contact with the base over the whole joint area, is sufficient to explain this behavior without invoking the structure of the melt. Note also that on heating the ribbon crystallizes well before melting (at the temperature T_x_), so any heredity effect, if real, must survive the solid-state transformations that precede melting.

For nickel systems it has been established experimentally that stable amorphization of ribbon up to 70 μm thick is achieved at a total metalloid content of no less than 20–25 at.% [68,69]. Deviation from the eutectic and alloying with chromium rapidly narrow the processing window; quantitative data are given in Section 7.1 [18,68,70]. Note that the very linkage “deep eutectic, glass former, and filler metal in one composition” was formulated for nickel systems as early as Rabinkin’s reviews [5,18], while criteria C1–C5 (Section 2.2) generalize it into a cross-system framework covering all the families of this review, including the negative controls. An analogous regularity is observed in titanium systems. The binary alloy Ti50Cu50 (at.%), corresponding to the best glass-forming composition of the Ti–Cu system (which is explained within the [Ti9Cu6]Cu3 cluster model [71]), amorphizes into ribbon 35–45 μm thick. The transition to multicomponent Ti–Zr–Cu–Ni compositions in accordance with the confusion principle [37] raises the critical thickness to 60–70 μm and beyond, and with additional alloying by Sn, Co, Fe, and Al the amorphous state is retained even when the total Cu and Ni content is reduced to 12 at.% [72]. According to the confusion principle, increasing the number of components with different atomic radii raises the crystal-chemical complexity of the melt, suppressing the nucleation and growth of competing crystalline phases and thereby promoting amorphization.

### 2.2. Criteria for the Applicability of an Amorphous System as a Brazing Filler Metal

Generalizing the experience of developing and industrially applying rapidly quenched filler metals, one can formulate five criteria that an amorphous system must satisfy simultaneously. Failure to meet even one of them either rules out the system’s use in brazing altogether or relegates it to the rank of niche solutions. The criteria are not new physical laws. They synthesize the selection rules established for the individual families [5,18,19,20,21] into a single cross-system checklist, as explained in the Introduction. They are also not strictly binary. Their fulfillment is graded (fully met, met with reservations, not met; Section 8) and depends on the particular composition and application. In Section 2.3 and Section 8 they are therefore applied to representative commercial compositions rather than to families as a whole. The criteria were derived retrospectively from the accumulated industrial experience and are validated in Section 2.3 and Section 4 against borderline and negative cases. Their expectation concerning phosphorus-bearing iron ribbons (Section 9) is so far consistent with the first commercial powders and research foils of this type, but has not been tested prospectively.

The temperature criterion (C1). The melting range of the filler metal must lie below the temperature threshold of degradation of the base material. For classical austenitic stainless steels (of the AISI 304 and 321 type) the brazing temperature should not substantially exceed 1100 °C, above which recrystallization sets in, while 1140–1170 °C serves as the absolute limit for most steels and nickel alloys [70,73]. For titanium alloys the limitation is the temperature of the polymorphic α → β transformation (883 °C for unalloyed titanium); for zirconium alloys, the analogous α → β transformation (863 °C for unalloyed zirconium) [51].

The function-coincidence criterion (C2). The melting-point depressant must simultaneously be a glass former, so the system must possess a GFA sufficient to produce continuous ribbon 20–60 μm thick at the cooling rates realized in industrial melt spinning (10^5^–10^6^ K/s) [24,25]. C2 is formulated for the amorphous state proper, in accordance with the scope defined in Section Scope and Literature Search Methodology. The rarer case in which rapid quenching yields a ductile ribbon without vitrification (Ni–Si–Be, Al–Ge–Si) does not satisfy C2 but may satisfy C4; it is treated below under C4 and in Section 4.3 and Section 7.4 as a boundary case of the class.

The base-material compatibility criterion (C3). This covers wetting and spreading over the base, limited erosion, and an acceptable phase composition of the seam. The depressants that provide fusibility and amorphization form brittle phases in the seam (chromium borides, nickel and iron phosphides, the intermetallics Ti_2_Cu and Ti_2_Ni) [29], and their amount must be controllable by the brazing schedule, up to nearly complete diffusional dissolution by the isothermal solidification (TLP) mechanism [20].

The processability criterion (C4). The ribbon must be ductile enough for the stamping of preforms of complex shape, must be windable into a coil, and must be producible in the required width (the industrial standard is 20–80 μm in thickness, with widths up to 50–200 mm from different manufacturers [53,60]). Note that C4 is not fulfilled automatically once C2 is, and the difference between them is a practical one. C2 means that the alloy amorphizes on quenching in principle, at least into a narrow laboratory ribbon a few millimeters wide. C4 demands more. Industrial casting to a width of 50–200 mm is a separate engineering task, since the stability of the melt puddle on a wide nozzle and the uniformity of thickness across the entire width have to be worked out separately. Zirconium alloys are especially telling. Their melt is so reactive that on heating it wets the material of the casting nozzle and the metal stops flowing out of the crucible [74], while at small amounts of oxygen in the chamber atmosphere the ribbon oxidizes immediately after leaving the quenching wheel. For this reason, producing calibrated zirconium ribbon even 20–30 mm wide remains a separate technological challenge (on the oxygen sensitivity of zirconium melts and ribbon casting see [75,76]; high-quality Metglas Ti–Zr ribbon initially came in widths of only up to 12.5 mm [19]). Moreover, metalloid-containing ribbons embrittle on relaxation annealing and prolonged aging long before crystallization. The classic example is the Fe–B–Si–C iron glass, which after low-temperature relaxation loses its ability to bend through 180 degrees [77]; among filler-metal systems, phosphorus-bearing ribbons are noticeably more sensitive to embrittlement than boron–silicon ones [78]. An alloy that formally passes C2 may therefore, with time, cease to survive the stamping of preforms. The reverse case is demonstrated by the Ni–Si–Be system. It does not amorphize, yet the same melt spinning yields a ductile nanocrystalline ribbon from it [79,80]. Ribbon as a form is thus attainable without vitrification, provided the phases formed on quenching are ductile, so the ability to amorphize (C2) and the processability of industrial ribbon (C4) constitute distinct selection axes.

The economic and safety criterion (C5). This comprises the cost of the components and toxicological restrictions. It is this criterion, and not the physics of glass formation, that excludes the palladium- and platinum-containing glasses with record GFA and restricts the use of beryllium-containing compositions.

For the purposes of this review the criteria are applied with the operational thresholds collected in Table 2. These thresholds are proposed by the authors. They are not taken from any standard, and the numerical values summarize the industrial practice cited in the table rather than sharp physical boundaries. Where a different application calls for different limits, the table can be re-populated without changing the logic of the framework. Two points deserve comment. First, the cooling rate of 10^5^–10^6^ K/s that appears under C2 is not a property of the alloy. It is the range realized by industrial single-wheel melt spinning for ribbon 20–80 μm thick [25], and it is the same for all systems. What distinguishes the systems is the critical thickness of ribbon that can be obtained fully amorphous at this rate. Second, the thresholds are deliberately graded into three levels (met, met with reservations, not met), and it is in these three grades that the criteria are applied in Section 2.3 and Section 8.

The independence of C2 and C4 can be shown systematically rather than by individual examples. Table 3 places the systems of this review in the four combinations of the two criteria; all four cells are populated. The upper-left cell is the industrial core of the class, the upper-right cell contains glass formers whose ribbon is difficult to cast wide or loses ductility on aging, the lower-left cell contains ribbons obtained without vitrification (outside the formal scope of the review, Section Scope and Literature Search Methodology), and the lower-right cell contains the compositions that exist only as powder or paste.

Criterion C3 deserves separate comment, since this criterion underlies one of the principal modes of application of amorphous ribbons, namely TLP bonding [29]. The small and precisely controlled ribbon thickness (20–60 μm) limits the amount of depressant in the brazing clearance, so that its diffusional outflow into the base metal is completed within technologically acceptable holding times, and the seam solidifies isothermally as a solid solution free of brittle eutectic constituents. The amorphous ribbon is thus not merely a convenient product form but a carrier particularly well suited to the TLP process, because the chemical homogeneity of the ribbon ensures that isothermal solidification proceeds uniformly over the entire joint area, something difficult to achieve with powders and pastes [5,23]. The exception is composite powder mixtures of filler metal with base-metal powder, which make it possible to conduct TLP brazing of wide gaps [29]. For nickel systems the TLP kinetics are limited, contrary to a widespread belief, not by boron but by silicon, whose diffusion coefficient in steel is substantially smaller [87], whereas for titanium systems TLP schedules make it possible to remove the intermetallics Ti_2_Cu and Ti_2_Ni from the seam. The common mechanism and its limits are set out in Section 2.4, and the family-specific features in Section 5.4 and Section 7.5. At the same time, TLP is not the only mechanism for forming a serviceable joint. In the brazing of ceramics and graphite, diffusional dissolution of the depressants into the base material is impossible, the seam retains its intermetallic structure, and the decisive factors become wetting with the formation of a reaction layer (TiC, TiN, Ti_5_Si_3_) at the interface, together with matching the elastic characteristics and coefficient of thermal expansion (CTE) of the brittle seam to those of the brittle base material [81,88].

Ribbon is not the only rapidly quenched product form. All the families considered can also be produced as powders obtained by melt atomization. The cooling rates in gas atomization (10^3^–10^5^ K/s) are lower than in ribbon melt spinning, so the powder structure depends on particle size: fine fractions have time to amorphize, whereas coarser ones come out amorphous-crystalline. Powders serve as the basis of pastes and composite mixtures, including those for TLP brazing of wide gaps, and complement ribbon where placing a foil in the gap is impossible for design reasons.

### 2.3. Systems That Passed the Selection, Borderline Systems, and Screened-Out Systems

The application of the formulated criteria to the principal known families of amorphous alloys is commented on below through characteristic examples, while the summary table and the brazing-temperature vs. base-material map in Section 8 close the comparison.

The systems whose representative commercial compositions meet all five criteria form the core of the commercial amorphous filler-metal product range. The assessment below and in Section 8 refers to these representative grades rather than to the families as a whole. The reason is that the fulfillment of C1 and C3 in particular varies within a family with composition and application, and the criteria are graded rather than binary (Table 2). Cu–Ni–Sn–P (MBF-2005, STEMET 1101) serves copper and its alloys, and on steels C3 is met only with reservations. Ti–Cu–Ni and Ti–Zr–Cu–Ni (BTi-1, BTi-3, STEMET 1202) are suitable for brazing titanium alloys, titanium intermetallics, and ceramics. For them C1 is fully met only by the compositions brazed below the α → β transformation and with reservations by the rest, and C5 is met with reservations for the beryllium-bearing STEMET 1202. Zr–Ti–Cu–Ni ribbons (STEMET 1409 and its analogs) are used for the zirconium alloys of nuclear engineering, as well as for titanium alloys and the active brazing of ceramics. For this family C5 is met with the reservation that its lowest-melting compositions contain beryllium, which requires special handling in production. Ni–Cr–Si–B and Ni–Cr–P (BNi-2, BNi-7, STEMET 1301) meet all five criteria for brazing stainless steels and heat-resistant nickel alloys, with the reservation that on steels the phosphorus grades are limited by the brazing clearance rather than by the hold (Section 7.5). Each of these families (copper–phosphorus, titanium, zirconium, and nickel) is considered in detail in the subsequent sections.

Of particular analytical interest are the borderline cases, that is, systems screened out by a single criterion, since these demonstrate the operation of the criteria in pure form. A representative example is the alloy BNi-5 (Ni–19Cr–10Si). It is a sought-after boron-free filler metal that nevertheless does not amorphize, because its only metalloid is silicon, whose content is insufficient for glass formation. As a result, BNi-5 is produced only as powder and paste, whereas its boron-containing counterpart BNi-5a (Ni–19Cr–7.3Si–1.5B; about 20 at.% Si and B in total versus roughly 18 at.% Si in BNi-5, boron being substantially more effective than silicon as a glass former [49,68]) is available as amorphous ribbon. The Ni–Si–Be system offers a second version of the same scenario: the filler metal exists, yet amorphization is unattainable; at moderate alloying (Si < 5, Be < 3.5 wt.%) the high ductility of the alloy allows a rapidly quenched ribbon to be produced in the nanocrystalline state [79,80]. The case of the Fe–Si–B alloys is instructive. The glass-forming ability of this system is among the best of the metal–metalloid alloys, and the system underlies the mass production of soft-magnetic ribbons. However, its brazing temperature of 1200–1220 °C falls outside the temperature window of steels (criterion C1) [29,89]. Only phosphorus-containing iron filler metals with brazing temperatures around 1100 °C are beginning to bring the system back among the candidates [13,14,89]. The industrial grades F300 and F302 are produced as gas-atomized powder for pastes [13,14,90], but the glass-forming ability of phosphorus-bearing iron melts is known from the classical Fe–P–C glasses [91], which is why precisely this branch is named in Section 8 as a candidate for a new ribbon family. Finally, Ti–Zr–Be and Zr–Cu–Be amorphize robustly, since beryllium is one of the strongest glass formers when added to Ti and Zr. They provide melting temperatures that are record-low for active filler metals (690–720 °C) and are indispensable in the brazing of SiC ceramics, graphite, and refractory metals. However, the toxicity of beryllium (criterion C5) keeps them in the status of small-batch solutions for nuclear and special-purpose engineering [88,92,93,94].

The operation of criterion C3 also shows up in attempts to apply the families beyond their “native” base materials. Thanks to their high reactivity, titanium and zirconium amorphous ribbons wet steels without flux, but brazing leads to the formation of brittle intermetallics of the TiFe and TiFe_2_ type in the seam. The hardness of such zones is high, and Ti-alloy/steel joints fracture in a brittle manner along the seam–steel boundary (see Section 5.6 for details) [95,96,97]. Similarly, copper–phosphorus ribbons form brittle iron phosphides when brazing steels [17]. In both cases the joint does form and is hermetic, but its use is limited to non-critical parts, as witnessed by the targeted development of Cu–Ni–Sn–P compositions with reduced phosphorus content, which lower the fraction of the brittle phosphide phase and the working brazing temperature [98]. On the map in Section 8 such cases are flagged with a separate “limited application” marker.

Systems screened out by several criteria at once are not used in brazing, and it is useful to record them as a negative control. BMGs of the Vitreloy type (Zr–Cu–Al–Ni–Be) possess outstanding GFA, but their use in brazing is limited first and foremost by the high beryllium content and the cost of the components. Only isolated demonstration studies of BMGs as brazing filler metals are known [99,100], and they confirm the rule. Aluminum-based amorphous alloys with rare-earth and transition metals (Al–Y–Ni, Al–La–Ni, Al–Y–Ni–Co) have no inexpensive element combining the depressant and glass-former functions, unlike B in the Ni–B filler-metal system or Cu in the Ti–Cu system. The noble-metal glasses Pd–Ni–P and Pt–Cu–P are excluded by cost, cheaper functional analogs being available.

### 2.4. Transient Liquid Phase Bonding with Eutectic Ribbons: The Common Mechanism and Its Limits

Three of the four families of this review, the titanium, zirconium, and nickel ribbons, are used on metallic base materials that dissolve their depressants. For all three the same joint-formation mechanism operates: transient liquid phase (TLP) bonding, also called diffusion brazing or isothermal solidification [29,101,102]. Its phase-diagram basis is the same in every case. It is conveniently read on a schematic eutectic diagram between the base metal and the depressant, such as those drawn for the Ti–(Cu + Ni) and Ni–(B + Si) systems in Section 5.4 and Section 7.5. The ribbon has a near-eutectic composition and melts completely at the brazing temperature, which lies above the eutectic and, for the multicomponent industrial ribbons, above the liquidus of the filler metal. Three stages follow. In the dissolution stage the melt dissolves the base metal until its composition reaches the liquidus point C_L_ at the brazing temperature. At this point the liquid layer is at its widest. In the isothermal solidification stage the depressant diffuses from the liquid into the base metal. A solid solution of the solidus composition C_S_ grows from both boundaries, and the solid/liquid interface advances toward the seam center while the liquid retains the composition C_L_. If the hold is interrupted, the remaining liquid solidifies on cooling into a brittle eutectic at the seam center. In the homogenization stage the depressant concentration in the solidified seam is levelled by solid-state diffusion, and the remelt temperature of the joint rises toward the solidus of the base metal. What differs between the families is only the identity of the depressant, its diffusivity and solubility in the base, and the phases that form when the depressant is bound rather than dissolved. These are intermetallics of the Ti_2_Cu and Ti_2_Ni type in the titanium and zirconium systems (Section 5.4) and chromium borides and nickel silicides in the nickel systems (Section 7.5).

Isothermal solidification is conveniently described as a diffusion-controlled process [29,101]. The depressant leaves the liquid interlayer for the base metal, and the solid/liquid interface advances into the seam according to a parabolic law:

X(t) = 2K√(D_s_·t) (5)

where X is the position of the solidification front, D_s_ is the diffusion coefficient of the depressant in the solid base metal, t is the holding time, and K is a dimensionless coefficient determined by the depressant concentrations at the interphase boundary. Since the front advances in proportion to √t, the total time of isothermal solidification of the seam grows in proportion to the square of the width of the liquid interlayer:

t_IS_ = π·W_max_^2^/(16·D_s_) · [C_Lα_/C_αL_]^2^ ∝ W_max_^2^/D_s_ (6)

where W_max_ is the maximum width of the liquid interlayer (the initial ribbon plus the dissolved base metal), and C_Lα_ and C_αL_ are the depressant concentrations in the liquid and in the solid solution at the interphase boundary, corresponding to the liquidus and solidus of the phase diagram.

Here, t_IS_ denotes the time of complete isothermal solidification of the liquid interlayer. The numerical prefactor is model-dependent, so what carries practical value is above all the scaling law itself, t_IS_ ∝ W_max_^2^/D_s_. Three consequences follow from it. First, the solidification time depends quadratically on the interlayer thickness, so a thin rapidly quenched ribbon 25–50 μm thick ensures complete isothermal solidification within tens of minutes to a few hours, depending on the depressant and temperature, whereas for thicker powder interlayers of the same mass it stretches out considerably. Second, the process rate is inversely proportional to the diffusion coefficient of the depressant: the small boron atom diffuses in the lattice of the base metal interstitially and orders of magnitude faster than the substitutional silicon and phosphorus [103,104,105]; moreover, the rate of isothermal solidification is governed by the product of the depressant’s diffusion coefficient and its solubility in the base metal [106], which explains why boride nickel systems solidify isothermally more readily than silicon- and phosphorus-containing ones (Section 7.5). Third, fully completed isothermal solidification removes the low-melting phase from the seam, raising the remelt temperature of the joint to the solidus of the base metal.

Equation (6) rests on the standard assumptions of the analytical TLP models [29,101,107,108]: one-dimensional diffusion of a single depressant into a semi-infinite base metal; a planar solid/liquid interface; local thermodynamic equilibrium at the interface, so that the concentrations C_Lα_ and C_αL_ are fixed by the liquidus and solidus at the brazing temperature; a composition-independent diffusion coefficient D_s_; a liquid that remains homogeneous; and lattice diffusion only, without a contribution from grain-boundary transport. W_max_ refers to the width of the liquid after the dissolution stage, not to the initial ribbon thickness. Under these assumptions the model is valid for metallic base materials with a finite solid solubility of the depressant, for gaps below the maximum brazing clearance, and for holding times up to the completion of isothermal solidification. Grain-boundary diffusion [109] and multicomponent effects [87,106] accelerate or retard the process relative to the prediction, which is why the numerical prefactor is not used in this review and only the scaling t_IS_ ∝ W_max_^2^/D_s_ is retained.

The mechanism is common, but it is not universal, and its limits deserve to be stated explicitly. First, the TLP description applies to joints in which the depressant can be dissolved by a metallic base with a finite solid solubility for it. At a ceramic or graphite surface the depressant cannot leave the seam through that interface; in a metal/ceramic joint it can still drain into the metallic side, but at the ceramic the seam retains its intermetallic structure and the joint is formed by a reaction layer (Section 5.5). The TLP description therefore does not cover the ceramic side of such joints. Second, within the metallic systems the mechanism operates fully for the titanium, zirconium, and nickel families. For the copper–phosphorus family it is metallurgically feasible, since phosphorus is both soluble and mobile in copper, but it is not practiced industrially and is not described in TLP terms in the literature, for the reasons discussed in Section 3.3. Third, the phosphorus-bearing nickel ribbons on steels are a special case. Phosphorus is removed from the seam not by diffusion into the base but by the growth of an iron-phosphide reaction layer, so the brazing clearance rather than the hold becomes the controlling parameter (Section 7.5). Fourth, for the zirconium family the mechanism is established qualitatively, but its kinetics have not been measured with the completeness available for the nickel and titanium systems (Section 6.1). When TLP is called the common mechanism of the class in the remainder of this review, it is in this qualified sense.

## 3. Copper–Phosphorus Amorphous Brazing Filler Metals

### 3.1. Copper Filler Metals and the Place of Phosphorus Systems

Copper brazing filler metals are used for brazing copper and its alloys, molybdenum, nickel, and galvanized steels, with the automotive industry (radiator and plate heat-exchanger production) as the largest consumer [17,110]. The ISO 17672 [16] standard divides copper filler metals into four groups: low-alloyed compositions (Cu, Cu–Ag, Cu–Ni, Cu–Sn; working temperatures of about 900–1100 °C), Cu–Zn brasses (870–920 °C), special alloys (Cu–Si–Mn, Cu–Al, Cu–Mn–Ni; 985–1075 °C), and the copper–phosphorus CuP classes (Cu–P, Ag–Cu–P; 645–825 °C). Pure copper melts at 1083 °C, while Cu–Sn tin bronzes have a solidus of about 875 °C and a liquidus of about 1025 °C [16]. The wide use of the CuP class stems from its self-fluxing ability: at a phosphorus content of 5–7 wt.%, brazing of copper in air requires no flux. The term “self-fluxing” is, however, conventional. Phosphorus does not form a flux in the classical sense; it acts as an active deoxidizer, reducing copper and nickel oxides, and it is this reducing ability that substitutes for a flux. On iron, the same phosphorus forms brittle phosphides, so the mechanism does not transfer to steels. The flip side is the low corrosion resistance in seawater and sulfur vapors, low high-temperature strength, and brittleness inherent in the class [17]. Most of the systems listed above are ductile and are rolled conventionally, so the amorphous niche within the copper family is confined precisely to the phosphorus-containing eutectic compositions, which is consistent with the logic of Section 2, since phosphorus here is simultaneously a melting-point depressant, a fluxing agent, and a metalloid glass former.

### 3.2. Amorphous Cu–Ni–P and Cu–Ni–Sn–P Systems

Binary Cu–P filler alloys have a comparatively high liquidus temperature (714–925 °C). Lowering it with silver (to 645 °C) makes the filler metal more expensive, while lowering it with cadmium raises environmental concerns. The answer to the cost of silver and the toxicity of cadmium came in the form of the silver-free Cu–Ni–P and Cu–Ni–Sn–P systems. The glass-forming ability of the binary Cu–P eutectic is modest: reliable amorphization is limited to ribbon thicknesses of 20–35 μm (see also [67,111]). Moving to the ternary and higher-order Cu–Ni–P and Cu–Ni–Sn–P systems, in line with the confusion principle (see Section 2.1), noticeably widens the processing window, and the critical amorphization thickness rises to 50–60 μm. Amorphous Cu–(7–30)Ni–(15–20)P (at.%) ribbons with a liquidus of 670–820 °C are described in patent US4209570A, and a Cu–Ni–Sn–P system with a zinc addition (at least 0.5 at.%, and up to 5 at.% for improved corrosion resistance in air) and a liquidus of about 650 °C in patent US7654438B2. Well-known commercial representatives are the amorphous Metglas MBF-2005/2007 ribbons (Cu76Ni6Sn5P13 at.%, T_liq_ = 655 °C) [58] and the OKC600 ribbon (CuNi4.2Sn15.6P5.3, 610 °C) of the CuproBraze technology for copper–brass radiators [112]; their Russian counterparts are the 11XX-series alloys of MIFI-AMETO LLC. These products are cited not as a catalog but as the evidence behind criterion C4: ribbon of this system has been cast industrially, in widths suitable for stamping, by at least three independent manufacturers. Standard ribbon thicknesses are 25–50 μm, the eutectic phosphorus content ensures reliable amorphization, and against the criteria of Section 2 the family passes the selection without reservation, except for criterion C3 on steel base metals.

Contact of a phosphorus-containing melt with iron and nickel generates brittle phosphides, and the classical rule forbids brazing Fe- and Ni-based alloys with copper–phosphorus filler metals [17]. Practice is more permissive than the rule: for non-critical parts, steels are brazed with Cu–P compositions as well, the embrittlement of the seam being accepted as a tolerable price for the low cost and simplicity of the process. This niche has been recognized by developers, as evidenced by the reduced-phosphorus compositions Cu–7Ni–4Sn–5P and Cu–10Ni–2Sn–6.5P created deliberately for brazing stainless steels, where shifting the balance from phosphides toward the copper solid solution improves seam ductility [113]. On the map in Section 8, these cases are flagged with a “limited application” marker, by analogy with titanium ribbons on steels. A separate branch is formed by the amorphous active Cu–Ti filler metals (25–50 wt.% Ti) for ceramic brazing [114,115], which are formally copper-based but functionally belong to the active titanium family of Section 5 and illustrate how arbitrary the boundaries between the families are.

### 3.3. Applications of Copper-Based Amorphous Filler Metals

The niche of copper–phosphorus amorphous ribbons is mass-produced heat-exchange equipment, namely copper–brass radiators (the CuproBraze technology), refrigeration and air-conditioning, and plumbing joints. The field remains active: for example, a Cu–Ni–Sn–P amorphous ribbon was recently used to braze porous copper to a copper substrate for heat-exchange structures [116]. The family wins through the lowest brazing temperature among amorphous filler metals (650–700 °C), the reducing ability of phosphorus, and the low cost of its components, but loses in the corrosion resistance and high-temperature strength of the joints. TLP kinetics on copper base metals have been studied less than on nickel and titanium ones and are of independent research interest, all the more so because the diffusional mobility of phosphorus in copper is high, which is in principle favorable for isothermal solidification [29]. At the same time, despite parameters favorable for TLP bonding (a solubility of P in solid copper at the eutectic of about 1.7 wt.%, an order of magnitude higher than the solubility of B in nickel, together with the high diffusional mobility of phosphorus), brazing with copper–phosphorus filler metals is virtually never described in the literature in TLP terms, in contrast to the nickel and titanium systems of this review. No metallurgical prohibition is evident here. With extended dwell times, the brittle Cu_3_P phase in copper/copper seams brazed with a Cu–Ni–Sn–P ribbon partially dissolves [117]; that is, isothermal dissolution of the phosphides is in fact observed, although the authors of such studies do not frame it in TLP terms. In our interpretation, which we offer as a hypothesis rather than as an established fact, the reason is historical rather than metallurgical. Cu–P technology evolved in plumbing and heat-exchange equipment, where a short dwell and a residual brittle eutectic containing the (Cu,Ni)_3_P phase in the seam are an acceptable price for the simplicity and low cost of the process, as the strength data presented below show. Both the term and the practice of deliberate isothermal solidification, by contrast, took shape in aerospace materials science, which works predominantly with nickel and titanium filler metals.

Quantitative data confirm these regularities for copper-to-copper brazing. For the serially produced filler metal STEMET 1101 (Cu–8.8Ni–7P–3.5Sn), the shear strength of copper/copper joints is 160–180 MPa [53]. For the compositionally similar STEMET 1105 (Cu–5Ni–6P–9Sn), the tensile strength of the same joints is 158 MPa [53], consistent with the independently obtained value of 135 MPa for the amorphous filler metal Cu68.5Ni15.7Sn9.3P6.5 [118]; in both cases, the strength is limited by the volume fraction of the brittle (Cu,Ni)_3_P phase in the seam. The brazing clearance remains the governing parameter here. For classical (non-amorphous) copper–phosphorus filler metals, the recommended clearance on copper tubing is 0.025–0.127 mm. When the clearance is larger, seam strength can be preserved only by switching to the more ductile silver-bearing compositions, such as BCuP-5 (Cu–15Ag–5P per AWS A5.8 [15]). This is exactly where the advantage of a thin amorphous ribbon manifests itself: its precise and thickness-stable geometry allows the minimum brazing clearance to be fixed directly during joint assembly. For STEMET 1101, the optimum assembly clearance of telescopic joints is 0.025–0.05 mm [53]. For copper–phosphorus eutectics this is a benefit, since a minimum clearance reduces the melt volume and thereby the fraction of the brittle phase in the seam. For the widely used industrial copper–zinc and copper–tin filler metals, outside the capillary range (above ~0.1–0.2 mm) gap filling becomes incomplete, porosity grows, and joint strength drops noticeably compared with the optimum narrow clearance. In the terms of the framework, these data document criterion C3 for copper base metals, with the joint strength limited by the phosphide fraction and hence by the clearance. The grading “met with reservations” on steels in Section 8 rests on a different observation, the formation of brittle iron phosphides when steels are brazed with these ribbons (Section 2.3), and not on the copper data.

An example of the seam structure is shown in Figure 3 for a copper joint brazed with a Cu–Ni–Sn–P amorphous filler metal at 680 °C [116]. The seam consists of a copper solid solution and a continuous phosphorus-rich reaction product (Cu–P phases) with nickel-enriched regions; phosphorus and nickel remain concentrated in the seam, whereas tin dissolves in the copper [116]. The same phase set, Cu(Sn,P) solid solution with Cu_3_P and Ni_2_CuP, was found in copper joints brazed with STEMET 1101 and STEMET 1105 at 650–700 °C and in the crystalline ingots of these alloys [56]. The residual phosphides show that the depressants have not been removed from the seam by diffusion into the base; the final microstructure alone does not establish how far isothermal solidification proceeded (Section 2.4). The phosphide fraction, which grows with the phosphorus content of the ribbon and decreases with brazing temperature and hold time, is what limits the toughness of the joints [56]. The seam obtained from the amorphous ribbon is also more uniform than that obtained from a cast filler of the same composition [52], which is the experimental basis of the statement, made in the Introduction, that the homogeneity of the ribbon is transferred to the seam. The different crystallization onset of ribbon-derived and ingot-derived melts [56] is discussed in Section 2.1 as an open question.

## 4. Aluminum and Silver Filler Metals and the Applicability Boundary of the Amorphous Form

Aluminum and silver filler metals constitute two major industrial families that formally fall outside the scope of the present review, since they are not produced as amorphous ribbons. The reasons for their absence from the amorphous roster are, however, not accidental and follow directly from the criteria of Section 2. First, for these systems the very motivation for amorphization disappears, because the ductile alloys are rolled conventionally [33]. Second, their base systems have low glass-forming ability. Examining these two families thus serves as a negative control for the remaining sections.

### 4.1. Aluminum Alloys

Aluminum brazing imposes a stringent temperature constraint, since the filler metal must melt below the base material (the melting point of aluminum is about 660 °C), and the standard Al–Si filler metals with a eutectic at 577 °C operate within a narrow window [119]. Aluminum–silicon filler metals are produced mostly as rods. A ribbon of the Al–Si eutectic can be obtained by casting; it is noticeably less flexible than a typical amorphous ribbon and fails only under substantial bending, while remaining sufficiently processable to be wound into a coil [84,85]. In the binary Al–Si system, the amorphous phase does not form at all, in contrast to the glass-forming Al–Ni–REM systems, and under criterion C2 the system remains outside the class of amorphous ribbon brazing filler metals. Even when an aluminum filler metal is produced by rapid quenching, the goal is a thin, uniform, ductile ribbon rather than a glassy state. The rapidly quenched Al–Ge–Si ribbons developed by the present authors for brazing dissimilar steel/aluminum joints have a microcrystalline rather than amorphous structure [84,85,86]. The authors’ own experience with rapidly quenched aluminum filler metals covers the Al–Si–Cu and Al–Si–Mg systems and the Al–Ge–Si system [83,84,85,120]. X-ray diffraction shows that these ribbons are microcrystalline, not glassy, and their properties were studied in the same way as those of the amorphous ribbons. Most of them are reasonably flexible, pass the reverse-bend test of criterion C4 (Table 2), and are used in the crystalline state. Rapid quenching here serves to refine the structure and to obtain a thin uniform ribbon, not to vitrify the alloy.

A peculiarity of aluminum is that amorphous aluminum alloys do in fact exist and are actively studied: these are the Al–REM–transition-metal systems (Al–Ni–Y, Al–Y–Ni–Fe, Al–Y–Fe, and the like). They are, however, high-strength structural materials whose value lies in a structure of aluminum nanocrystals embedded in an amorphous matrix, providing strengths of 1400–1700 MPa [121]; that is, they were developed for mechanical rather than joining tasks. The reasons for their unsuitability as filler metals under criteria C1 and C2 are analyzed in Section 4.2 together with the analogous case of silver alloys.

### 4.2. Silver Filler Metals and Ductility Instead of Amorphization

Silver filler metals (Ag–Cu–Zn, Ag–Cu–In, Ag–Cu–P, Ag–Cu–Sn, as well as the active Ag–Cu–Ti compositions considered at the end of this section) form the most versatile family in brazing as a whole. The Ag–Cu eutectic melts at 779 °C [51], and alloying with zinc, tin, indium, and cadmium lowers the melting range to 600–650 °C. The property of these alloys most relevant to the present review is their high ductility, thanks to which they are rolled into foil and drawn into wire by conventional methods and therefore simply have no need for rapid quenching as a route to thin ribbon. This is a direct manifestation of the logic of Section 2: amorphization by melt spinning is a forced solution for brittle eutectics, and silver filler metals are not brittle.

The second reason is metallurgical: silver systems have low glass-forming ability. The binary Ag–Cu system amorphizes poorly; attempts to obtain a metallic glass from the low-melting Ag–Cu–Ge and Ag–Cu–Sb eutectics mostly failed [122]; bulk silver glasses have been found only in exotic multicomponent systems of the Ag–Mg–Ca–Cu type; and in ternary Cu–Zr–Ag alloys, the amorphous state does not form at all once the silver content exceeds 35 at.%. Thus, even if a technological need existed, obtaining an amorphous silver ribbon would be difficult.

A telling example is patent US4182628, which describes a partially amorphous Ag–Cu–In foil (Ag 25–66, Cu 16–68, In 7–18 at.%). Normally, as shown above, silver alloys are ductile and are rolled without rapid quenching; however, at an indium content above 12 wt.% the composition ceases to be processable: rolling no longer yields a ductile foil. The same mechanism operates here as in the main families of this review: rapid quenching suppresses the formation of brittle crystalline phases, and the alloy retains ductility in the amorphous/metastable microcrystalline state. Amorphization becomes necessary precisely when ordinary crystallization produces a brittle structure, regardless of the underlying alloying system.

A special place among silver filler metals belongs to the active compositions of the Ag–Cu–Ti system. This is a broad class of active brazing filler metals differing in titanium content; typical representatives include Cusil-ABA, Ticusil, and others. Titanium provides wetting and chemical bonding to non-metallic surfaces, making Ag–Cu–Ti the classical solution for joining dissimilar materials such as ceramics, glasses, graphite, and other brittle materials not wetted by conventional filler metals. They are supplied both as rolled ribbons (foils) and as powders and pastes. Like the other silver filler metals, Ag–Cu–Ti alloys are ductile, are rolled by conventional methods, and have no need for the amorphous ribbon form, although functionally they solve the same active-brazing task as the amorphous titanium and zirconium ribbons.

### 4.3. Splitting of Criterion C2 as the General Lesson from Aluminum and Silver

Aluminum and silver exhibit a characteristic splitting of criterion C2 of Section 2, which combines the depressant and glass-former functions. In terms of the scope defined in Section Scope and Literature Search Methodology, rapidly quenched but non-amorphous ribbons are outside the class: they satisfy C4 (a ductile ribbon is obtained) without satisfying C2, which is precisely why the two criteria are kept separate. The ordinary ductile filler metals (Ag–Cu–Zn, Al–Si) contain a suitable depressant (Cu–Zn and Si, respectively) and satisfy temperature criterion C1, but their base systems have low glass-forming ability and do not amorphize. That is, the “depressant” half of C2 is fulfilled, while the “glass-forming” half is not. For the compositions with genuinely high GFA (Ag–Mg–Ca–Cu, Al–Ni–Y, Al–Ni–Ce, Al–Fe–Y, and others), the situation is reversed: amorphization is possible for them (the “glass-forming” half of C2 is fulfilled), but their alloying elements do not act as a practical depressant for brazing. Criterion C1, moreover, is violated in both directions: for Ag–Mg–Ca–Cu the melting temperature is anomalously low (T_m_ ≈ 350–380 °C according to [123]), the level of soft solders rather than structural brazing, whereas the Al–Ni–Y alloys are, on the contrary, high-melting, and their melting temperature leaves no processing window for brazing aluminum structures. Criteria C3 and C4 fare no better. Calcium and magnesium are chemically active, oxidize readily, and evaporate on melting in air, which hampers stable melt spinning of a ribbon of constant thickness (criterion C4) and creates a risk of oxide contamination of the seam during brazing (criterion C3). Yttrium and gadolinium, as rare-earth elements, are reactive and prone to forming brittle intermetallics at the interface with the base material, further reducing compatibility under criterion C3. The cost and limited availability of rare-earth elements, together with the environmentally problematic technology of their mining and separation, additionally rule such compositions out under criterion C5. Thus, neither silver nor aluminum compositions with glass-forming ability sufficient for amorphization satisfy criteria C1–C5 simultaneously. This failure across all criteria at once explains why silver- and aluminum-based alloys are not used industrially as amorphous ribbon brazing filler metals. Table 3 (Section 2.2) places these cases alongside the other systems of the review in the four combinations of C2 and C4 and shows that the aluminum and silver examples are not isolated: ribbon without vitrification and glass without an industrial ribbon both occur in several families.

## 5. Titanium-Based Amorphous Brazing Filler Metals

### 5.1. Classification and the Base Ti–Cu System

Titanium-based brazing filler metals are used in aerospace engineering, shipbuilding, petrochemistry, medicine, and power engineering; they are distinguished by high activity, good fluidity, and a combination of strength and corrosion resistance in the brazed joints [20,124]. By alloying principle, titanium filler metals fall into two groups. The first is formed by Ti(–Zr) systems with eutectic-forming depressants (Cu, Ni, Fe, Co, Be), which sharply lower the melting temperature but form hard, brittle intermetallics with titanium. Such alloys are unsuitable for rolling and drawing and are produced as thin foils up to 70–80 μm thick exclusively by ultrarapid quenching (melt spinning), or as powders obtained by milling annealed ribbon or by melt atomization. The second group consists of solid-solution alloys of Ti with refractory bcc metals (Nb, Ta, V, Cr), which retain ductility and can be rolled conventionally. They are considered only briefly in Section 5.7, since the amorphous state is neither required nor achievable for them, in full analogy with the solid-solution Ni–Mn filler metals of Section 7.4.

The base eutectic system of the first group is Ti–Cu. The composition Ti50Cu50 (at.%, corresponding to roughly 57 wt.% Cu) exhibits the best glass-forming ability among the binary alloys of this system, which is explained within the short-range-order cluster model [Ti9Cu6]Cu3 [71]. Nevertheless, the GFA of the binary system remains moderate: no glass transition is resolved before crystallization in binary Ti–Cu glasses [125,126], and the critical thickness of the amorphous ribbon is 35–45 μm [52,76]. For the titanium and zirconium ribbons the parameters ΔT_x_ and γ are therefore not available, as for the copper and nickel ribbons of Table 1; what is measured is the crystallization onset (about 425 °C for STEMET 1203 and about 500 °C for STEMET 1201, DTA at 20–40 K/min [52]) and the critical thickness (35–45 μm for the binary and 50–70 μm for the Ti–Zr–Cu–Ni ribbons [52,76]). Commercial binary-based ribbons are produced in Russia, namely STEMET 1203 (Ti–50Cu) and STEMET 1204 (Ti–72Cu), wt.% [28]. The Ti–Cu phase diagram contains several eutectic points. On the titanium side lies the eutectic L → β-Ti + Ti_2_Cu (about 1005 °C, 29 at.% or 36 wt.% Cu). It is followed by the eutectic L → Ti_2_Cu + TiCu (960 °C, 43 at.% or 50 wt.% Cu) and by the eutectic in the copper corner at 875 °C (73 at.% or 78 wt.% Cu); the Cu-rich side of the diagram contains a cascade of phases TiCu_2_, Ti_2_Cu_3_, Ti_3_Cu_4_, and TiCu_4_. The glass-forming range spans 30–78 at.% Cu, that is, 36–83 wt.% [51]. Besides Ti_2_Cu, which is responsible for embrittlement of the seam, the diagram includes the compounds TiCu and TiCu_4_, and the solubility of titanium in copper reaches 8 at.% (6 wt.%) at 885 °C. Remarkably, the position of the GFA maximum cannot be reduced to eutectic depth alone. The composition Ti50Cu50 amorphizes markedly better than Cu–28Ti (~72 wt.% Cu, near the third, copper-corner eutectic point) [71,127], which points to the decisive role of the specific short-range order of the [Ti9Cu6]Cu3 cluster [71] rather than of the thermodynamic depth of the eutectic per se. Crystalline (non-amorphous) eutectic Ti–Cu alloys were used for brazing titanium in foil and powder form long before the advent of rapid quenching; the transition to the amorphous ribbon form is associated with the wave of titanium–zirconium Metglas filler-metal development in the early 1990s [19], in the wake of which the present-day binary ribbons also appeared. The Ti–Cu melt wets a titanium base almost perfectly, with a contact angle close to zero, owing to the chemical affinity of the melt for the titanium base and the solubility of copper in β-Ti, behavior typical of active and kindred systems [81].

### 5.2. The Ti–Cu–Ni System

Joint alloying with copper and nickel yields the Ti–Cu–Ni family of filler metals, standardized by AWS as BTi-1 (Ti–15Cu–15Ni, melting range 910–960 °C) and BTi-2 (Ti–15Cu–25Ni, 890–940 °C); the BTi titanium group has been present in AWS A5.8M/A5.8 [15] since the 2011 edition and is retained in the current 2026 edition, whereas ISO 17672 [16], including its current 2024 edition, contains no titanium class. These are the most widely used titanium filler metals in world practice. Under the trade names Ticuni and Ticuni-60 (Morgan Advanced Materials), Ti-flo 950 (Johnson Matthey), TiBraze15-25 (Titanium Brazing), ANA 3 (Prince & Izant), and Meta-Braze BTi-1/2 (VBC Group), they are supplied as powders, pastes, stamped preforms, and amorphous ribbons of the MBF-5000 series (Metglas). They are used for brazing commercially pure titanium and alloys of the VT1-0 type (foreign analog TA2), as well as the two-phase (α + β) alloy VT6 (Ti–6Al–4V, foreign analog TC4), honeycomb structures, and heat exchangers; the brazing temperature of 970–1050 °C lies above the α → β transformation, which requires that recrystallization of the base metal be taken into account. The microstructure of Ti/Ti seams produced with Ti–Cu–Ni filler metals is multiphase: alongside α/β-titanium, the seam contains the intermetallics Ti_2_Cu, Ti_2_Ni, and mixed Ti_2_(Cu,Ni), and, in zirconium-containing systems, the still more complex (Ti,Zr)_2_(Cu,Ni), whose amount and morphology are governed by the brazing temperature, time, and clearance [128]. A high concentration of intermetallics embrittles the seam and reduces the shear and tensile strength, so controlling their fraction is the central task of the brazing schedule and leads directly to the TLP approach (Section 5.4).

### 5.3. The Ti–Zr–Cu–Ni System and Multicomponent Compositions

The introduction of zirconium, an element with unlimited solubility in titanium that simultaneously lowers the melting temperature, yields the quaternary Ti–Zr–Cu–Ni system, which is optimal in its combination of low melting temperature, glass formation, and joint properties. The standard compositions are Ti–37.5Zr–15Cu–10Ni (BTi-3, also known as TiBraze375, 825–843 °C) and BTi-5 (Ti–20Zr–20Cu–20Ni, 848–863 °C), while the MBF-5000 series of amorphous ribbons (Metglas) covers compositions from Ti–15Zr–15Cu to Ti–25Zr–50Cu [19]. Brazing of commercially pure titanium and Ti–6Al–4V with these compositions (Ti–37.5Zr–15Cu–10Ni, Ti–35Zr–15Cu–15Ni, Ti–25Zr–50Cu) was investigated as early as the classic study [129]. The glass-forming ability of the quaternary system is markedly higher than that of the binary one (ribbon 50–70 μm thick is obtained reliably [19,130]), and with multicomponent alloying by Sn, Co, Fe, and Al the amorphous state is retained even when the total Cu + Ni content is reduced to 12 at.% [72,131].

The effect of composition on the properties of titanium-alloy joints follows a pattern that is consistent across the studies compiled in [20], although the magnitude of each effect depends on the composition range and the brazing schedule. Increasing the zirconium content strengthens the seam by the solid-solution mechanism, whereas raising the total concentration of the depressants Cu + Ni reduces the strength because of the Ti_2_Cu and Ti_2_Ni intermetallics. Additional alloying pursues two goals: lowering the brazing temperature below the α → β transformation (883 °C) so as to preserve the structure and properties of the base metal, and increasing the ductility of the seam. Silver (Ti–12.5Zr–13.7Cu–11.6Ni–2.2Ag, complete melting at 820 °C) raises the strength and impact toughness of the joints relative to silver-free analogs [132,133]; tin stabilizes the supercooled liquid and lowers the melting temperature [134]; cobalt and iron [135], palladium [136], and vanadium [137] regulate the diffusion processes and phase composition; vanadium, in particular, reduces the amount of intermetallics in the seam and increases ductility. The record low-melting composition of the family, 35Ti–25Zr–30Be–10Co with complete melting at 750 °C, already belongs to the beryllium branch.

Beryllium is the most effective depressant known for Ti–Zr systems: within the composition range of the filler metals considered here, each weight percent of Be lowers the liquidus temperature by roughly 150 °C [92,93]. This opens up compositions melting at 750–950 °C (Ti–48Zr–4Be, 35Ti–25Zr–30Be–10Co) and at the same time, because beryllium is a strong glass former, ensures, at Be contents above 3 wt.%, the production of thin ductile foils in the amorphous state. The beryllium addition improves fluidity, which is important for capillary brazing of thin-walled structures. The principal niche of Ti–(Zr)–Be is the brazing of refractory metals (Mo, Nb, W, Ta), graphite, ceramics, and composites, chiefly in nuclear-industry products. A telling example is the fully low-activation composition 48Ti–48Zr–4Be, developed for joining tungsten, tantalum, and low-activation steel for the DEMO project [93]. The metallurgical case for beryllium is thus strong. Its limitations are of two different kinds, one metallurgical and one regulatory, and they should be kept apart. First, beryllium has low solubility in most refractory metals, and brittle beryllides form in the seams, so that fracture of joints in the steel/Ta/W pair proceeds through the intermetallic Ta_2_Be [93], while TiBe_2_ is observed in seams with ceramics [88]. Second, the toxicity of beryllium (criterion C5 of Section 2) requires special measures in ribbon production and keeps the family in the status of small-batch solutions for special-purpose engineering.

Beyond titanium alloys, titanium-based filler metals are used for joining the titanium aluminides TiAl and Ti_2_AlNb to heat-resistant superalloys [138,139,140], as well as in dissimilar pairs with steels and nickel alloys; the latter cases entail the formation of brittle intermetallics and are treated separately in Section 5.6.

### 5.4. TLP Brazing with Titanium Ribbons

For titanium systems, the TLP approach solves the intermetallic problem in the same way that it solves the boride–silicide problem for nickel systems. When the holding time exceeds the depressant dissolution time, copper and nickel diffuse from the melt into the titanium base metal, which has substantial solubility for both elements, and the seam solidifies isothermally as an α/β-titanium solid solution with a minimal content of Ti_2_Cu, Ti_2_Ni, and more complex intermetallics. A clear illustration is the evolution of a Ti/Ti joint brazed with the 50Ti–25Zr–10Cu–15Ni filler metal at 880 °C: after a 10 min hold the seam contains eutectoid colonies with intermetallics, whereas after 60 min it becomes an almost homogeneous solid solution [128,141]. Under optimal schedules the joint strength reaches the level of the base metal, and for Ti–6Al–4V joints increasing the brazing temperature and time monotonically raises the shear strength through homogenization of the seam [136,142]. The staged mechanism of this process is illustrated schematically in Figure 4.

The mechanism has been established on wedge-shaped specimens, which allow the maximum brazing clearance (MBC) to be assessed, for the pairs OT4 (a pseudo-α titanium alloy of the Ti–Al–Mn system)/STEMET 1202 (Ti–12Zr–22Cu–12Ni–0.8V–1.5Be ribbon) and VT6-s/STEMET 1202 [141]. The process proceeds in three stages (Figure 4). First comes dissolution of the base metal by the melt. The liquid composition thereby shifts toward point 1 on the liquidus, corresponding to equilibrium of the liquid with the β phase at the brazing temperature. Diffusional isothermal solidification follows. The depressants (Be, Ni, Cu) leave the liquid for the base metal, a β-phase layer of the composition of point 2 grows from the seam boundaries, and the liquid/solid interface moves toward the seam center; the liquid retains the composition of point 1. As the depressants drain away, the composition of the β phase at the boundary with the base metal enters the two-phase α + β region, and α-plates of the composition of point 4 grow within the β phase (Widmanstätten structure), the β phase at their boundary corresponding to point 3. Solid-state homogenization completes the process. If the holding time and temperature are insufficient, a brittle eutectic forms on cooling from the residual liquid at the seam center (zone III), surrounded by a eutectoid zone (zone II) and a Widmanstätten structure (zone I, the strongest). The strength of joints containing eutectic in the seam is only 200–550 MPa, whereas at 840 °C for 2 h the eutectic disappears from the seam completely and the strength reaches the level of the base metal. It amounts to 747–754 MPa for OT4 and 902–909 MPa for VT6-s, with fracture proceeding through the base metal.

The kinetics of dissolution and isothermal solidification in the titanium–copper pair have been investigated quantitatively on model joints with a pure copper interlayer [143]. Both depressants, Cu and Ni, have high diffusion coefficients in β-titanium, which makes the titanium TLP process markedly faster than the nickel one, which is rate-limited by silicon (Section 7.5). Of particular practical value are TLP schedules below the α → β transformation temperature (883 °C), realized with the lowest-melting multicomponent ribbons, since they make it possible to combine a solid-solution seam with preservation of the initial structure and mechanical properties of the titanium base metal [141]. Only the lowest-melting compositions stay below this threshold, for example STEMET 1202 (catalog brazing window 850–900 °C; 840 °C for 2 h in the TLP experiment [141]), whereas the standard BTi-1 and BTi-5 are brazed above the α → β transformation.

The kinetics of dissolution and isothermal solidification are described by Equations (5) and (6) in Section 2.4, together with the assumptions of the model; only the titanium-specific features are considered here.

### 5.5. Active Brazing of Ceramics and Graphite

The high affinity of titanium for oxygen, carbon, and nitrogen makes titanium-containing (as well as zirconium-containing) amorphous ribbons active filler metals that wet non-metallized ceramics, graphite, and composites without prior surface preparation [81]. Interaction with the substrate proceeds through the formation of reaction layers, whose type is determined by the class of ceramic: titanium and zirconium carbides and silicides on silicon carbide materials (SiC); nitrides and silicides/aluminides on nitride ceramics (Si_3_N_4_, AlN); titanium and zirconium oxides on oxide ceramics (Al_2_O_3_, ZrO_2_); and, on graphite and diamond, Ti and Zr carbides formed directly without the participation of silicon. The thickness and continuity of the reaction layer are controlled by the brazing temperature and time; its excessive growth, as in metallic pairs, leads to embrittlement, since the reaction products are very hard, and such joints fracture through the reaction layer at loads below 100 MPa [144].

A metal’s propensity for active brazing of ceramics is governed by the Gibbs energy of formation of its compounds with the nonmetallic constituents of the ceramic: oxides on oxide ceramics, carbides on carbide ceramics, graphite, and diamond, and nitrides on nitride ceramics: the more negative ΔG° of the corresponding compound, the stronger the thermodynamic driving force for the metal to react with the ceramic surface and wet it. A representative measure of this activity is the affinity of the metal for oxygen, quantified by the Ellingham diagram, since the metals that form the most stable oxides also form stable carbides and nitrides. In this series, titanium and zirconium occupy one of the extreme positions among technically usable metals, yielding only to the alkaline-earth metals and aluminum, whereas copper, nickel, and iron form substantially less stable compounds and do not wet non-metallized ceramics without the introduction of an active element (Figure 5).

The diagram also implies a limitation that matters specifically for oxide ceramics. The Ti line lies below the Cu_2_O, NiO, Cr_2_O_3_, B_2_O_3_, and SiO_2_ lines but above the Al_2_O_3_ and ZrO_2_ lines, and the Zr line lies below the same metal-oxide lines and coincides with the ZrO_2_ line by definition. The active components therefore confidently reduce oxide films on the metals being brazed and silica-containing phases, whereas complete reduction of alumina and zirconium dioxide by titanium is thermodynamically impossible under standard conditions, and for zirconium against zirconia the standard comparison gives no driving force at all. A comparison of the standard lines, however, does not describe the real interfacial reaction: it concerns the standard formation reactions of the selected stoichiometric oxides and does not by itself determine the interfacial reactions in a multicomponent braze, where component activities, oxygen dissolution in the metal, and oxide non-stoichiometry must also be considered. In the studies of alumina brazing cited here, the product of the interaction of titanium with oxygen at the interface with the ceramic is reported to be not stoichiometric TiO_2_ but the suboxides TiO and Ti_2_O_3_ and ternary oxides of the Ti_3_(Cu,Al)_3_O type; notably, the TiO line lies distinctly below the TiO_2_ line and approaches the Al_2_O_3_ line to within 15–20 kJ/mol O_2_ (Figure 5). The aluminum released by partial reduction dissolves in the melt, where its activity is far below unity, which shifts the equilibrium toward the reaction; the titanium activity in the melt, likewise below unity, works in the opposite direction, and the outcome is determined by the balance of these contributions. As a result, partial reduction with dissolution of oxygen in titanium is thermodynamically allowed at the interface with alumina, and wetting is provided by the forming reaction layer of titanium suboxides and ternary oxides [81]. Gettering of the residual oxygen of the furnace atmosphere by titanium and zirconium plays an auxiliary role, maintaining a low oxygen potential and clean surfaces, but by itself it does not explain wetting. For zirconia-based ceramics, reduction in the usual sense is not defined, since the filler-metal element and the ceramic cation coincide; the interaction reduces to partial reduction to non-stoichiometric ZrO_2−x_ with dissolution of oxygen in the seam metal, accompanied by the characteristic darkening of the ceramic. To nitride ceramics (Si_3_N_4_, AlN) the oxide diagram does not apply, and their wetting is governed by the reactions forming titanium nitrides and silicides.

The demonstrated range of applications is broad and includes brazing of zirconia-based ceramics to the 29NK alloy with the amorphous ribbon STEMET 1202 (Ti–12Ni–12Zr–22Cu–0.8V–1.5Be, melting range 750–815 °C [53]) [146]; active brazing of silicon carbide with rapidly quenched TiZrCuBe [88]; joining of ZTA (zirconia-toughened alumina) ceramic to titanium for endoprosthetics [147]; brazing of molybdenum to graphite with Ti–Zr–Nb–Be powder for X-ray tube anodes, with endurance thermal testing [148,149]; joining of a Cf/SiC composite to 304 steel with a Ti–Zr–Be filler metal [94]; and brazing of sapphire to titanium alloys. The main quality factors are CTE matching of the materials being joined, where the ductile constituent of the seam plays a damping role, and precise dosing of the active element; here the advantages of a homogeneous amorphous ribbon over powders manifest themselves in full.

### 5.6. Use of Titanium Alloys for Brazing Steels and Their Limited Applicability

The activity of titanium filler metals makes it possible to braze steels without flux, and formally steel/steel and Ti-alloy/steel joints can turn out sound in the absence of significant residual stresses. However, the formation of the intermetallics TiFe and TiFe_2_ in the seam is unavoidable, and in contact with austenitic steels the σ phase and Ti(Cu,Ni)_2_ phases form as well. The microhardness of such zones reaches 1100 HV, and fracture proceeds in a brittle manner along the seam–steel boundary [95,96]. The strength of dissimilar Ti-alloy/steel joints turns out to be 30% or more below that of all-titanium ones, and the strength-versus-schedule dependence has a pronounced optimum, since increasing the temperature and time simultaneously improves wetting and thickens the intermetallic layers [138]. The possibilities for improvement are limited and reduce to optimizing the zirconium content of the ribbon [95], to copper or nickel barrier interlayers that interrupt the Ti–Fe contact, and to lowering the brazing temperature. The practical conclusion for the present review is that, for steels, titanium ribbons remain a material of limited applicability, suitable for Ti/steel transition joints and non-critical parts (the “limited applicability” marker on the map in Section 8), whereas for steel structures proper the nickel systems of Section 7 remain the main choice, with the iron-based ribbons of Section 7.7 as an emerging alternative.

### 5.7. Product Range, Manufacturers, and the Solid-Solution Branch

Foreign production of titanium filler metals is concentrated in a small number of large companies. Titanium Brazing, Inc. (Hilliard, OH, USA) produces the TiBraze grades as powders and amorphous ribbons, including the niobium-containing TiBraze200Nb/920 and the high-entropy TiBraze1215 of composition Ti60Cu10Ni10Mo10Co10 at.% (the first commercial high-entropy alloy (HEA) filler metal); Metglas manufactures the MBF-5000 series of amorphous ribbons; Morgan Advanced Materials produces Ticuni; Johnson Matthey produces Ti-flo; VBC Group produces Meta-Braze; and Prince & Izant offers full product ranges. In Russia, amorphous ribbons are produced by MIFI-AMETO LLC, among them STEMET 1201 (Ti–12Ni–12Zr–24Cu), STEMET 1202 (Ti–12Ni–12Zr–22Cu–0.8V–1.5Be), STEMET 1216 (Ti–22.5Cu–9Ni–12.5Zr), STEMET 1228 (Ti–23Zr–17Cu–15Ni, nominal composition within the manufacturer’s specification [53]), as well as the binary STEMET 1203 (Ti–50Cu) and STEMET 1204 (Ti–72Cu), wt.%. Typical products include honeycomb structures made of TA2, radio-electronic device housings, and heat exchangers made of VT1-0 [27].

The solid-solution branch Ti–(Zr)–bcc-metal (Ti–30V, Ti–33Cr, Ti–35V, Ti–10Nb, and others, brazing temperatures of 1350–1750 °C) closes the classification of Section 5.1. These rollable foils are intended for brazing refractory metals, with joint temperature capability up to 1400–1800 °C (after diffusional saturation of the seam with the refractory base metal, the remelt temperature of the joint can exceed the melting temperature of the original filler metal) [150], and they do not require the amorphous state. Their existence underscores the central thesis of this review: rapid quenching is needed precisely where depressants render the alloy brittle.

To summarize the section in the terms of Section 2: titanium-based amorphous ribbons are advantageous where rapid quenching does two things at once. It provides a low-melting, stampable filler metal from a composition that is brittle in the cast state (criteria C1, C2, and C4), and it suppresses the coarse intermetallics that the same depressants would otherwise form in the ribbon. The TLP hold can then remove much of the depressant-rich phase from the seam (C3). The limits of the family are equally clear. On steels the same reactivity produces TiFe-type intermetallics (C3 met only with reservations). The compositions brazed above the α → β transformation alter the structure of the base metal (C1 with reservations). The lowest-melting compositions rely on beryllium, whose toxicity keeps them in special-purpose engineering (C5).

## 6. Zirconium-Based Amorphous Brazing Filler Metals

### 6.1. Zirconium as a Filler-Metal Base

Zirconium is among the most chemically active transition metals. In its affinity for oxygen it surpasses virtually all transition metals and is second only to the alkaline-earth metals, being comparable to aluminum (Figure 5, Gibbs energies of oxide formation). However, the melting point of pure zirconium (1852 °C) requires intensive alloying to produce brazing filler metals. Two routes are used: solid-solution alloying with titanium, or eutectic alloying with the depressants Cu [151,152], Ni [153,154], Co [147], Fe [155], Al, Be [92,156], and even Pd [157]. Under a sufficiently clean furnace atmosphere, zirconium-based filler metals wet most metallic and non-metallic materials by reducing their surface oxide films, and they possess good fluidity and capillary activity; as shown below, the same reactivity makes their spreading sensitive to the oxygen potential of the atmosphere, to the surface condition, and to the superheat. When interacting with oxide, nitride, and carbon-containing materials, zirconium enters into reactions of the type Zr + O_2_ → ZrO_2_, 2Zr + N_2_ → 2ZrN, and Zr + C → ZrC. These reactions underlie its high activity [81].

The fluidity of zirconium-based fillers is strongly affected by their own ZrO_2_ surface film, which can delay droplet formation even above the liquidus temperature. It has been established experimentally that at moderate heating rates (5–10 °C/min) a droplet of zirconium-based filler begins to oxidize and lose its shape at a superheat of as little as 50–100 °C above the liquidus line. This occurs even in a vacuum of 10^−4^–10^−5^ mm Hg. Complete spreading over ceramic substrates is therefore not achieved, precisely because of oxidation [158]. Droplet-formation delay caused by the alloy’s own oxide film is also characteristic of zirconium-bearing silver-based fillers: for the AgCu5Zr ribbon (T_liq_ = 860 °C), a droplet was observed only at 1000 °C, i.e., at a superheat of ~140 °C. Separately, reducing the zirconium content also weakens the wetting activity itself: on switching to AgCu1.5Zr, the contact angle on Al_2_O_3_ ceramic rose to 65°, markedly poorer than for titanium-bearing analogs of the same matrix (11–25°) [158].

The principal advantage of zirconium-based fillers over titanium-based ones is the possibility of reaching lower complete-melting temperatures: in the Zr–Cu–Be, Zr–Cu–Ni–Be, and Zr–Cu–Fe–Be systems, ternary eutectics near the zirconium corner yield alloys with melting onsets below 700 °C (melting ranges of 673–711 and 690–720 °C). This makes it possible to braze zirconium and titanium alloys below the α → β phase-transformation temperatures, avoiding recrystallization and loss of the mechanical properties of the base metal, and simultaneously lowers the level of residual thermal stresses in the seams, which is critical when joining ceramics and dissimilar pairs (titanium–zirconium, zirconium–steel) [156,159]. From the standpoint of glass formation the family is well positioned, since deep multicomponent eutectics and the presence of beryllium, one of the strongest glass formers, ensure reliable production of ribbons. Zr–Be glasses have been known since the era of binary amorphous ribbons [92], and Zr–Ti–Cu–Be compositions underlie BMGs [160]. Product forms include amorphous ribbons, powders, and cast preforms.

Seam solidification during brazing of zirconium and titanium alloys with zirconium-based fillers in small gaps proceeds by the same TLP mechanism as for titanium ribbons; it is therefore not repeated here, and only a system-specific result is given. When Zircaloy-4 is brazed with a Zr–Ti–Cu–Fe filler below 900 °C, dissolution of the alloying components into the base metal does not go to completion, brittle eutectic is retained in the joint center, and the strength drops, whereas under optimal regimes the joint strength exceeds that of the base metal [155]. Copper and nickel are known to be anomalously fast diffusers in the bcc β phases of both titanium and zirconium, where their mobility exceeds the solvent self-diffusion by orders of magnitude, so rapid isothermal solidification is to be expected for both base metals. However, systematic measurements of TLP kinetics in zirconium systems comparable in completeness to those for nickel systems are absent from the literature, and the thesis of fast kinetics rests on isolated results and on the analogy with titanium ribbons. Thus, for the zirconium family as well, TLP is the mechanism by which a sound joint is formed, but its kinetics remain the least quantified of the four families, and the statements on rapid isothermal solidification in this family should be read as an expectation based on the analogy with titanium and on isolated results rather than as an established regularity (see also Section 2.4).

### 6.2. Nuclear Engineering: Applications and Corrosion Constraints

The principal niche of zirconium-based filler metals is reactor-core components and structures made of zirconium alloys (Zircaloy-4, E110), where welding is difficult or undesirable [21]. Demonstrated applications include brazing of VVER-440 spacer grids made of E110 alloy with the multicomponent filler STEMET 1410 (Zr–8Cu–2Sn–5.5Fe–1Nb–0.4Cr–(2.5–3.5)Be–(0.5–1)Ge, manufacturer’s specification [53]) [27,28], zirconium/stainless-steel transition joints [156], and joints of Zircaloy-4 to the MAX phase Ti_3_AlC_2_ [161]. For in-reactor applications a specific compositional restriction applies: the filler metals must not contain titanium, which sharply accelerates the electrochemical corrosion of brazed joints in zirconium alloys. Autoclave testing in water at 360 °C (80 h) shows degradation of seams brazed with the Zr55Ti25Ni20 filler, whereas the titanium-free Zr67Cu17Ni16 remains resistant [162]. This is a characteristic example of criterion C3 acting in its corrosion dimension, where a composition that is optimal in terms of melting behavior and glass formation is screened out by the service conditions.

### 6.3. Applications Beyond Nuclear Engineering

Their high activity makes zirconium ribbons fully fledged active filler metals for ceramics and graphite. Demonstrated applications include brazing of SiC ceramics with Zr–Cu and Zr–Cu–Nb fillers [151,152,163,164], ceramic feedthroughs for vacuum equipment, the ceramic femoral head of a hip endoprosthesis to a VT1-0 sleeve with a Zr–Ti–Co filler [147,165], and joining of zirconia ceramic to titanium [153]. A distinct historical niche is the brazing of niobium and its alloys for nuclear engineering with Zr–V–Fe, Zr–Nb–Be, and Zr–Ni compositions at brazing temperatures of 1050–1302 °C [76,150]; these efforts remained one-off in character, owing to the limited field of application of niobium alloys and the dominance of welding [166]. In dissimilar zirconium–titanium pairs, zirconium-based fillers provide corrosion-resistant joints [159]. The high-temperature oxidation resistance of the joints, however, is limited. Increasing the zirconium content of the filler degrades the oxidation resistance of the seam. Under isothermal holds in the range of 600–750 °C, the mass gain of specimens grows noticeably with increasing zirconium content.

### 6.4. Product Range and Status of the Family

The commercial product range of zirconium-based filler metals is the narrowest among the four amorphous families of this review. The only foreign supplier is Titanium Brazing, Inc. (Hilliard, OH, USA), with the amorphous ribbons TiBraze590 (Zr–17.5Ti–21.5Ni, 796–813 °C), TiBraze620 (Zr–11Ti–20Ni–7Nb, 791–808 °C), and TiBraze800 (Zr–15Ti–13Ni–7.5Cu, 901–915 °C) for brazing titanium, ceramics, graphite, and composites. In Russia, ribbons of the STEMET 14xx series are produced by MIFI-AMETO LLC (e.g., the STEMET 1409 alloy with a melting range of 690–750 °C and a brazing temperature of 750–850 °C [53]). The industrially demonstrated applications are confined to small-batch products (spacer grids of E110 alloy, zirconium/steel transition joints, ceramic feedthroughs). The joining of SiC, graphite, MAX phases, and biomedical ceramics discussed in Section 6.3 remains at the level of laboratory demonstrations, and the family has not seen wide industrial use; at the same time, brazing is regarded as a promising method for joining critical zirconium structures [21], and the combination of record-low melting temperatures, high activity, and the radiation compatibility of the titanium-free compositions is not reproduced by any other family.

In the terms of Section 2, the zirconium family is defined by two strengths and four practical boundaries. The strengths are the highest chemical activity of the four families and the lowest melting temperatures among the titanium and zirconium families. C1 is therefore met even below the α → β transformations of titanium and zirconium, and C2 is met thanks to multicomponent eutectics, in the lowest-melting branch with beryllium as a strong glass former. The first boundary is the oxidation sensitivity of the melt, which constrains both ribbon casting (C4 met with reservations) and spreading during brazing. The second is the corrosion constraint that excludes titanium from in-reactor compositions (C3 in its corrosion dimension). The third is beryllium: the lowest melting temperatures are reached with it, and its toxicity and the special handling it requires are what limit the lowest-melting branch under C5. The fourth is the limited industrial availability, with a single foreign supplier and small-batch production in Russia. The kinetics of TLP bonding in this family, finally, remain the least quantified of the four (Section 2.4 and Section 6.1).

## 7. Nickel-Based Amorphous Brazing Filler Metals

### 7.1. Nickel as a Filler-Metal Base

Nickel-based brazing filler metals form the most extensive and versatile family for high-temperature brazing of stainless steels and heat-resistant alloys, since their joints retain strength and corrosion resistance at service temperatures of 600–800 °C, whereas silver- and copper-based fillers are heat-resistant only up to 400–500 °C [70]. A systematic treatment of nickel filler metals for austenitic steels across all product forms (powders, pastes, ribbons), with a detailed examination of seam microstructure, TLP kinetics, strength, and corrosion, is given in the predecessor review of the present work [32]. To avoid duplicating it, this section considers the nickel family from the angle specific to the present review, namely through the ability of its systems to be converted into the amorphous ribbon state and through the place of nickel ribbons within the unified series of the four amorphous families. For details on steel brazing, TLP regimes, and corrosion behavior, the reader is referred to [32].

From the standpoint of Section 2, the key point is that all three depressants of the nickel systems (B, P, Si) are classical metalloid glass formers [167]. The Ni–Si–B and Ni–Cr–P systems amorphize into ribbon up to 70 μm thick at near-eutectic contents of boron (about 17 at.%, 3 wt.%) and phosphorus (about 19 at.%, 11 wt.%), and robust glass formation requires a total metalloid content of at least 20–25 at.%. Departing from the eutectic rapidly narrows the processing window. Lowering the metalloid content to 6 at.% B (1.2 wt.%) and 9 at.% P (5 wt.%) reduces the critical amorphization thickness to 30–40 μm. The introduction of chromium, necessary for corrosion resistance, likewise lowers the glass-forming ability, and at 22 at.% Cr (20 wt.%) the limiting ribbon thickness is 35–45 μm even at near-eutectic metalloid content. It is this regularity, rather than reference grade compositions, that distinguishes the present treatment from [32].

### 7.2. The Boron–Silicon Ni–Cr–Si–B and Ni–Si–B Systems

A number of the most widespread amorphous ribbons are built on the ternary Ni–Si–B eutectic, among them BNi-2, BNi-3, BNi-4 (AWS A5.8 [15]), the Russian STEMET 1301 (an analog of BNi-2, melting range 970–1020 °C [53]) [26], and the ribbons of the Asha Metallurgical Plant [168]. The difference in melting range between the compositionally close BNi-3 and BNi-4 has been analyzed in detail at the level of the monovariant lines of the Ni–Si–B system [32]. Shifting the composition away from the eutectic toward lower metalloid content simultaneously widens the melting range (which is convenient for brazing wide gaps) and lowers the glass-forming ability. The requirements of capillary brazing and those of amorphization thus point in the same direction, toward the eutectic, while wide-gap brazing conflicts with both. Alloying with chromium up to 10 wt.% further lowers the melting temperatures, and BNi-2 (T_liq_ = 1024 °C) serves as the de facto benchmark of the family, whereas above 10 wt.% Cr the liquidus rises (BNi-1, 1103 °C), which in grade VPr11 (Ni–15Cr–4Fe–4.5Si–2.5B–0.6C, amorphous ribbon and powder) is compensated for by carbon (1020 °C). The tungsten-bearing BNi-10, BNi-11, and VPr27 (for compositions see Section 7.4) are the most heat-resistant and possess a wide melting range for repair brazing.

The structure of the seam produced by brazing with nickel-based filler alloys containing silicon and boron is dual in nature. In the central part of a narrow gap (30–50 μm), boron and silicon have time to diffuse into the base metal and the seam crystallizes as a nickel solid solution, whereas in the fillet and at wide gaps (about 150 μm) the eutectic of the filler metal is inherited, with needle-like chromium borides and nickel silicides [169,170]. Figure 6 shows this for AISI 304L joints brazed at 1160 °C with Ni–Cr–Si–B foils of increasing chromium content [171]. The seam is the isothermally solidified nickel solid solution (ISZ), bounded on both sides by the diffusion zone (DZ) of the steel with fine chromium borides on the grain boundaries; with the longer hold the ISZ widens and the borides in the DZ coarsen, whereas in the 20 wt.% Cr joint blocky silicides and borides of the athermally solidified zone (ASZ) remain at the seam center, because chromium and molybdenum in the filler slow the outflow of boron and silicon into the steel [171]. The phases of such seams were identified by EBSD in [171] and by X-ray diffraction and electron-probe microanalysis in [31,169,170]. A detailed analysis of the phase composition of the seams, of the boride network in the diffusion-affected zone (DAZ), and of their effect on properties is given in [32]. What matters for the present review is that controlling the fractions of these phases is the task of the TLP regime, and that the amorphous ribbon, owing to the precise thin gap it sets and to its homogeneity, is a particularly suitable carrier for it.

### 7.3. The Phosphorus-Bearing Ni–Cr–P System

Phosphorus-bearing filler metals (BNi-6, BNi-7, BNi-12) stand out for their exceptional spreading, for the reducing action of phosphorus toward nickel oxides, and for the lowest brazing temperatures in the family. The ternary eutectic BNi-7 (Ni–14Cr–10P, T_m_ = 888 °C) flows freely into gaps down to 25 μm, providing hermetic joints in honeycomb structures and thin-walled tubes, while BNi-12 (25 wt.% Cr) was developed for enhanced resistance to oxidation and electrochemical corrosion. On solidification, part of the chromium is bound into mixed (Ni,Cr)_3_P phosphides and Cr–P-enriched phases [172,173], and no quantitative data on the effect of chromium on the isothermal solidification kinetics of the phosphorus systems are available in the literature. The production technology of phosphorus ribbons continues to be refined, for example by pre-deoxidizing the melt before casting [174]. Another route to reducing the fraction of brittle phosphides is alloying with copper, including the composite filler BNi-7 + 3% Cu [173,175]. The principal niche is nuclear applications, where boron, with its high neutron capture cross-section, is inadmissible [44]; the radiation stability of the phases in BNi-6 and BNi-7 seams has been verified experimentally under irradiation with 300 keV Ni^+^ ions [176]. The limitation of the system is the brittle phosphides that embrittle the seam [32], and the mechanism of their removal depends fundamentally on the base metal being brazed. Phosphorus diffuses into the base metal slowly because, like silicon, it moves by the substitutional (vacancy) mechanism, in contrast to interstitial boron [103,105], and its solubility in solid nickel is negligible (less than about 0.3 at.% even at the 870 °C eutectic [177]), so the base offers both a small diffusion flux and a small sink capacity. Nickel, moreover, forms its own stable phosphide Ni_3_P. Nevertheless, on nickel bases the isothermal solidification of Ni–P follows the classical TLP law. When nickel is brazed with a Ni–11P interlayer at 1150 °C, the width of the eutectic decreases linearly with the square root of time down to its complete disappearance, and the finer the grain of the base, the faster the process, a direct indication of the contribution of grain-boundary diffusion of phosphorus [109]. The phosphide phase in the seam therefore dissolves away markedly more slowly than the borides in the Ni–Cr–Si–B systems (slower, but not fundamentally differently), and the answer to embrittlement remains either long holds or control of the gap and brazing regime (on the MBC and the associated strength data, see Section 7.5 and Table 4).

From the glass-formation standpoint, the phosphorus compositions are favorable: the eutectic phosphorus content (about 19 at.%) is close to the canonical amorphizing metal–metalloid compositions, and Ni–Cr–P ribbon up to 60–80 μm thick is obtained reliably.

### 7.4. The Ni–Cr–Si, Ni–Mn, and Ni–Si–Be Systems

The nickel family provides several telling borderline cases illustrating criterion C2 of Section 2. The heat-resistant BNi-5 (Ni–19Cr–10Si, brazing temperature 1150–1200 °C), the second most widespread nickel filler metal and the standard choice for boron-free nuclear applications, does not amorphize, since silicon is its only metalloid and its content is insufficient for glass formation. BNi-5 is therefore produced only as powder and paste, whereas its boron-containing modification BNi-5a is available as ribbon, and the BNi-5/BNi-5a pair demonstrates criterion C2 in its purest form. An intermediate position is occupied by complex-alloyed heat-resistant compositions with refractory additions (Co, W, Mo, Nb) at 1.2–1.5 wt.% B and 6–7.5 wt.% Si. The Russian VPr42 and VPr50 are produced as powders, while their amorphous ribbon analogs have been brought into production by MIFI-AMETO LLC as STEMET 1342 (Ni–14Co–8.5Cr–6W–6.5Si–1.2B, an analog of VPr42, brazing at 1100–1150 °C) and STEMET 1350 (Ni–19Cr–9Co–5Mo–6Si–1.3B–1.2Nb, an analog of VPr50, brazing at 1140–1150 °C); STEMET 1309 (Ni–15Cr–4Fe–7.5Si–1.5B–4Mo, Section 7.5) belongs to the same group. Despite their low boron content, these compositions amorphize into ribbon 30–40 μm thick and occupy the same position on the glass-formation scale as BNi-5a [53]. VPr24, VPr32, VPr36, and VPr37 do not amorphize and exist only as powders.

The Ni–Mn system (grades G40NKh, G70NKh, VPr7, VPr8, as well as the near-eutectic BNi-8 with a silicon addition) provides minimal structural inhomogeneity of the seam and high strength, and manganese improves the spreading of the filler over steels. The compositions of these grades are given in Table 5. However, the solid-solution Ni–Mn alloys can be rolled into 80–100 μm foil by conventional methods and have no need of rapid quenching. Their application is limited by low corrosion resistance and by stringent demands on the purity of the brazing atmosphere, since manganese oxidizes more readily than chromium and the dew point must not exceed −57 °C [32].

The Ni–Si–Be system is instructive as a workaround with respect to criterion C2. Beryllium plays the role of a depressant much like boron, but it is highly soluble in nickel and does not provide glass formation. The ribbon obtained here is not amorphous but nanocrystalline. It is of good quality only at reduced contents of silicon (below 5 wt.%) and beryllium (below 3.5 wt.%), as in the alloy Ni–5Si–3Be (T_liq_ about 1130 °C). The lower-melting alloy Ni–6Si–5Be (915 °C) is brittle because of its high fraction of intermetallics. At the same time, joints of Kh18N10T steel made with such ribbons reach the strength level of the base metal (530 ± 50 MPa) [80,178], while the limitations of the family remain the oxidizability and toxicity of beryllium (criterion C5).

The Fe–Si–B alloy is the mirror case of BNi-5: its glass-forming ability is high, but its brazing temperature falls outside the admissible temperature window of steels (criterion C1), whereas the phosphorus-containing analogs that lower the brazing temperature do not themselves amorphize. The compositions, temperatures, and details of the iron filler metals are considered in Section 7.7.

### 7.5. TLP Brazing with Nickel Ribbons and Service Properties

TLP bonding is the technological answer to the main problem of nickel filler metals, the brittle boride, silicide, and phosphide phases, and its mechanism, kinetics, and effect on corrosion are analyzed in detail in [29,32,179,180]. Brazing is carried out at T_b_ = 1050–1070 °C, above the liquidus of the filler metal (1024 °C). The ribbon of eutectic Ni–Si–B composition (the red point in Figure 7, ≈25 at.% B + Si) melts completely. At the dissolution stage, the melt dissolves the base metal and the composition of the liquid shifts from that of the filler toward the point C_L_ on the liquidus. The width of the liquid layer is at its maximum at this point. Isothermal solidification then follows. A nickel solid solution of composition C_S_ grows from the boundaries with the base. Boron is practically insoluble in it, so during the hold it diffuses into the steel and is bound by chromium into borides, forming zones I–III. Silicon dissolves in nickel to a limited extent but diffuses into the base markedly more slowly than boron, so it is silicon that limits the rate of isothermal solidification and the required hold. The liquid retains the composition C_L_ while its fraction decreases to zero. If the hold is interrupted earlier, the remaining liquid crystallizes athermally on cooling into a brittle eutectic of borides and silicides at the center of the seam. On cooling, the solubility of the metalloids in nickel falls along the solvus, which can produce secondary precipitates within the seam itself. First and foremost, the thin homogeneous ribbon is a particularly suitable carrier for TLP, because its limited and precisely dosed reserve of depressant has time to diffuse into the base within acceptable holds, and the absence of segregation and of any binder ensures uniform completion of isothermal solidification over the entire joint area, which is difficult to achieve with powders and pastes, whose depressant reserve depends on the packing and binder content [5,23]. Moreover, TLP kinetics in the nickel systems are limited not by boron but by silicon, with its low diffusion coefficient [87,181]. The residual liquidus then lies on the silicon branch, and the closure of the solidification fronts is controlled precisely by the removal of silicon down to the solidus composition. Finally, TLP makes it possible to raise the remelt temperature sharply: the remelt temperature of joints made with eutectic BNi-9 exceeds 1370 °C after a diffusion hold [31,70].

The mechanism of boride network formation has been traced in detail on austenitic steel brazed with Ni–Cr–Si–(Fe)–B ribbons [31]. In the DAZ of the austenitic steel, three morphological boride zones are distinguished (Figure 7). The first adjoins the solid solution and contains fine chromium borides inside the original grains. In the second, borides cover most of the grain boundaries. In the third, borides remain only on isolated boundary segments. Reducing the boron content of the filler from 2.6 to 1.5 wt.% noticeably decreases the amount of borides, but further reduction is impossible. The glass-forming ability of a metal–metalloid system requires 15–30 at.% metalloids, and with insufficient boron the quality of the ribbon deteriorates. Raising the chromium content of the filler above that of the steel (22 wt.% versus 12–18% in the steels) keeps the chromium concentration in the diffusion zone at its initial level, but provokes the appearance at the seam center of a stable CrB-type boride phase that does not disappear even after a 4 h hold. The composition Ni–20Cr–7.5Si–4.5Fe–1.5B proved optimal in the overall balance of properties, giving the most homogeneous structure and a joint strength of 500 ± 40 MPa, whereas the original STEMET 1301A and the chromium-rich compositions give 340–430 MPa.

Notably, directed TLP brazing with Ni–P fillers in which isothermal solidification is driven to completion is practically undescribed in the literature for nickel alloys proper, including chromium-bearing ones, although model experiments prove that it is feasible in principle. On pure nickel, the isothermal solidification of a Ni–11P interlayer at 1150 °C obeys the square-root-of-time law and can be driven to complete disappearance of the eutectic, accelerating as the base grain size is refined [109], and on the heat-resistant alloy GH3039, raising the brazing temperature to 1150 °C thins the brittle centerline eutectic zone of Ni_3_P and raises the shear strength of the joints to 174 MPa, although within practical holds the eutectic does not disappear completely [182]. The reason this route has not seen industrial development apparently lies not in the kinetics but in the harmfulness of phosphorus to the nickel alloy itself. Unlike boron, which is sometimes deliberately introduced into nickel superalloys in trace amounts as a grain-boundary strengthener, phosphorus is a classical harmful impurity element that segregates to grain boundaries and degrades stress-rupture strength and creep resistance [183,184]. The eutectic in the Ni–P system forms at 880 °C, whereas the lowest-melting eutectic in the Ni–B system forms at about 1100 °C. This means that, when phosphorus-containing fillers are used and phosphorus diffuses along the grain boundaries into the base material, grain-boundary liquation can occur at considerably lower temperatures than with boron-bearing fillers. Even a kinetically complete removal of phosphorus from the seam therefore does not remove the concerns about the effect of its residual trace amounts on high-temperature properties.

In practice, the filler metals BNi-6 (Ni–11P) and BNi-7 (Ni–14Cr–10P) are used to join stainless steels in assemblies that are not load-critical, rather than to produce joints at the strength level of the base material. With steels, moreover, a fundamentally different mechanism operates: phosphorus reacts with the iron of the base to form an iron-phosphide reaction layer, which hinders further removal of phosphorus into the base material and embrittles the brazed joint.

The strength of joints produced with nickel filler metals depends on the filler composition, the brazing regime, and the morphology of the residual phases in the seam; for the three most widespread fillers this is summarized in Table 4. Corrosion damage of joints made with boron-containing ribbons begins in the diffusion zone, which is depleted in chromium owing to the formation of chromium borides. Raising the chromium content of the filler to 13–18 wt.% (as, for example, in the filler STEMET 1309 of composition Ni–15Cr–4Fe–7.5Si–1.5B–4Mo, wt.%) prevents the depletion and improves resistance to intergranular corrosion and corrosion fatigue [169,185,186].

**Table 4:** Room-temperature tensile strength of butt-brazed AISI 316 stainless steel joints as a function of the filler metal and the brazing clearance (data of Lugscheider and Partz [187], as compiled in [32]).

Filler Metal and Brazing Regime	Tensile Strength at the Optimum Clearance	Tensile Strength at Clearances Above the MBC
BNi-2 (Ni–Cr–Si–B); 1040 °C, 10 min	≈500–540 MPa (base-metal level)	≈50 MPa (below 10% of the base metal; brittle fracture through the boride eutectic)
BNi-5 (Ni–Cr–Si, boron-free); 1090 °C, 60 min	≈500–540 MPa (base-metal level)	350–400 MPa (silicide eutectic, less embrittling than borides)
BNi-7 (Ni–Cr–P); 1065 °C, 60 min	≈300 MPa (0.4–0.5 of the base-metal strength) in the regimes studied	100–130 MPa

Note: Butt-brazed specimens of Type 316 stainless steel with controlled clearances of 0–110 μm, vacuum-brazed and tested in uniaxial tension at room temperature [187]. The values are read from the strength–clearance curves reproduced in [32] and are rounded to about ±10 MPa, since the source reports single curves without scatter bands; the ±10 MPa is thus the accuracy of reading the compiled plot, not the scatter of the tests. The exact clearance corresponding to each value is not given in [32], and the regimes, including 1090 °C for BNi-5, are quoted as reproduced in [32], the primary report [187] not having been available to us. The maximum brazing clearance (MBC) is the largest clearance at which no eutectic remains at the seam center for the given regime. For BNi-2 and BNi-5 it increases with brazing temperature, whereas for BNi-7 it is nearly independent of it (Section 7.5). For comparison, room-temperature tensile tests of AISI 321 joints brazed with STEMET 1301A and its modified compositions (1160 °C, 15–40 min, clearance equal to the ribbon thickness) gave 370 ± 30 to 500 ± 40 MPa, with fracture along the steel/seam boundary [31].

The different behavior of BNi-2 (Ni–Cr–Si–B) and BNi-5 (Ni–Cr–Si) at gaps above the MBC, namely the drop of BNi-2 to ~50 MPa (<10% of the base metal, brittle) versus 350–400 MPa for BNi-5, is consistent with borides embrittling the seam more strongly than silicides in this comparison. Phosphorus-bearing BNi-7 in its best regime (narrow gap) reaches only ~300 MPa, i.e., 0.4–0.5 of the base-metal strength, and at gaps above the MBC it falls to 100–130 MPa. The reason lies in the kinetics of phosphorus removal and in its sink capacity. Phosphorus diffuses by the substitutional mechanism markedly more slowly than interstitial boron and is almost insoluble in the solid solution of the base, so on steels isothermal solidification proceeds slowly. Removal of phosphorus from the seam is provided not by direct diffusion into the base but by the growth of a dense iron-phosphide layer at the interface (diffusion of P to the steel/phosphide boundary and of Fe to the phosphide/melt boundary), a slow and self-decelerating process. The MBC of BNi-7 is therefore the narrowest in the family and, unlike that of BNi-2 and BNi-5, remains practically independent of brazing temperature [32]. For the phosphorus systems it is the gap, not the hold, that serves as the main controlling parameter in steel brazing: the solidification window cannot be widened by heating, as it can for the boron systems. As a consequence, the remelt temperature for the boron systems after a TLP hold can exceed 1370 °C, whereas for the phosphorus systems, if the centerline eutectic zone is retained (at gaps above the MBC), it is limited in principle by the Ni–P eutectic temperature of about 880 °C.

### 7.6. Applications and Product Range

The main application areas of nickel-based amorphous ribbons are honeycomb seals and assemblies of gas-turbine engines, plate and tubular heat exchangers (including exhaust gas recirculation (EGR) coolers), nuclear-engineering products (phosphorus-bearing boron-free compositions), and vacuum and cryogenic engineering. The brazeable base metals also include refractory metals. Molybdenum and tungsten are brazed with nickel filler metals, both with conventional boron–silicon compositions of the BNi-2 type and with the specialized tungsten-bearing BNi-10/BNi-11 for the most heat-resistant joints. Nickel filler metals also serve outside their native base materials: TiAl/CoCrFeMnNi high-entropy-alloy dissimilar joints have been vacuum-brazed with the nickel filler BNi-2 [188].

Abroad, the production of amorphous nickel ribbons is concentrated at Metglas (the MBF series), Vacuumschmelze (VITROBRAZE), VBC Group, Höganäs, Proterial, and others, while powder and paste forms are produced by Wall Colmonoy, Prince & Izant, and Morgan Advanced Materials. In Russia, nickel-based amorphous ribbons are produced by the Asha Metallurgical Plant (75N13KhSR, 82N7KhSR, 71N18KhSR, 92NSR, 80NKh15R) [168] and by MIFI-AMETO LLC (STEMET 1301 as an analog of BNi-2, STEMET 1309, and the heat-resistant STEMET 1342 and STEMET 1350), while powders are produced by VIAM (the VPr series, of which VPr24/32/36/37 are powder only) and Polema JSC. Adjacent to the nickel ribbons is the cobalt amorphous ribbon family Co–Cr–(Ni)–Si–B (BCo-1, Metglas MBF-80 ribbon) for heat-resistant cobalt alloys; owing to its narrow niche it is not considered in detail in this review, but in the matrix of Section 8 it occupies the same cell as the BNi ribbons.

**Table 5:** Filler-metal grades mentioned in Section 3, Section 5 and Section 7 but not included in the summary table in Section 8 (non-amorphizing, solid-solution, or narrowly specialized systems; compositions and temperatures from AWS A5.8M/A5.8 [15], the manufacturers’ catalogs [53,60], and [32,189]).

Grade	Composition, wt.%	T, °C	Product Form	Status/Application
BNi-10/BNi-11	Ni–12Cr–3Fe–3.5Si–2.5B–16W–0.5C/Ni–10Cr–3Fe–4Si–2.5B–11.5W *	970–1105	powder	heat-resistant; repair brazing of wide gaps
VPr27	Ni–9Cr–2.5Si–9W–1.5Nb–9Co–1.5Al	1031	powder	heat-resistant Ni alloys
VPr24, VPr32, VPr36, VPr37	heat-resistant nickel systems Ni–Cr–Co–(W, Mo, Nb)–Si–B with reduced boron content	—	powder only	do not amorphize
G40NKh	Ni–9Cr–(6–8)Fe–40.5Mn–(0.10–0.25)B	1065–1130	rolled foil, 80–100 μm	solid-solution, non-amorphous
G70NKh	Ni–Cr–Si–70Mn (approximate)	~1030	rolled foil	solid-solution, non-amorphous
VPr7	Ni–0.4Cr–1Fe–0.1Si–33.5Mn–(2.0–2.5)Nb–10.5Co	1100–1120	solid-solution foil	steels
VPr8	Ni–0.5Si–33.5Mn–(4.0–9.5)Nb–(4.0–9.5)W–(10.0–11.5)Co	1140	solid-solution foil	heat-resistant alloys
F300	F300-10: Fe–20Cr–20Ni–7P–4Si–10Cu [90]	≥1120 [90]; 1100 in [14]	powder	nickel replacement, steels
F302	Fe–15Cr–10Ni–9P–5Si–5Cu	~1100	powder	steels
BCuP-2, BCuP-5 (crystalline Cu–P)	Cu–7P/Cu–15Ag–5P	710–795/645–800	rod, strip	classical self-fluxing fillers for copper; ductile only with silver; not amorphous (Section 3)
Ti–30V, Ti–33Cr, Ti–35V, Ti–10Nb	solid-solution Ti–bcc-metal alloys	1350–1750	rolled foil	refractory metals; ductile, amorphous state neither needed nor achievable (Section 5.7)

* For the tungsten-bearing BNi-10 and BNi-11, amorphization is possible in principle, but no data on the critical ribbon thickness were found in the literature; the amorphization depth is probably small, and in practice these compositions are produced as powder.

### 7.7. Iron-Based Brazing Filler Metals

Iron-based brazing filler metals deserve separate consideration. Unlike the nickel-based ones, they have not gained wide industrial adoption and are used, as a rule, to solve local problems in the design of one-of-a-kind equipment. The reason is the combination of brittleness, difficulty of manufacture, and the high melting temperature of the base compositions, typically on the order of 1200 °C. To lower the melting temperature, silicon, boron, and phosphorus are introduced into iron filler metals, while tungsten and chromium are added to improve high-temperature oxidation resistance and high-temperature strength. Because of their low ductility, such alloys are most often used as powder or paste rather than ribbon, which sets them noticeably apart from most of the systems considered in this review. Nevertheless, interest in iron filler metals has been growing in recent years, driven by the high cost of nickel and by tightening environmental requirements on industry [13,189].

Iron filler metals are conventionally divided into boron-containing and phosphorus-containing ones. The former include compositions of the Fe–5.3Si–3B and Fe–12Cr–7.5Si–2B type (wt.%), whose brazing temperature lies in the range 1200–1220 °C [29,89]. The latter include the industrial gas-atomized powders F302 (Fe–15Cr–10Ni–9P–5Si–5Cu [14]) and F300 (variant F300-10: Fe–20Cr–20Ni–7P–4Si–10Cu, recommended brazing temperature 1120 °C and above according to the manufacturer’s data sheet [90]; 1100 °C in the study [14]), for which the presence of phosphorus lowers the brazing temperature relative to the boron-containing iron fillers, while phosphorus itself additionally improves the wetting and spreading of the filler over the base material [14]. At the same time, the corrosion resistance of joints made with phosphorus-containing iron fillers in acidic media is markedly higher than that of their boron-containing iron and nickel counterparts, though this is achieved at the cost of lower seam strength [190].

A notable example is the rapidly quenched filler metal Fe–12Cr–Si–B developed for brazing the reactor steel EP-823 in the BREST-OD-300 project [191]. Nickel is excluded here as a matter of principle: it forms a low-melting eutectic with the lead coolant at 324 °C and corrodes in it. The resulting amorphous–crystalline ribbon 40–45 μm thick (T_m_ 1070–1150 °C) provided a brazed-seam strength of 750 ± 90 MPa, comparable to the base metal. The amorphization thickness achieved is comparable to the nickel analogs: low-chromium near-eutectic Ni–Cr–Si–B compositions amorphize at ribbon thicknesses of 60–70 μm, and chromium-rich compositions with 20 wt.% Cr at 35–45 μm; that is, the glass-forming propensity of this iron alloy is not substantially inferior to that of nickel analogs with a similar chromium content.

The nickel family can be summarized as a set of trade-offs that the framework of Section 2 makes explicit. Glass formation requires 20–25 at.% of metalloids (C2), and this is exactly what produces the brittle borides, silicides, and phosphides in the seam (C3). The conflict is resolved by the TLP hold, whose kinetics are set by silicon in the boron–silicon systems and by the iron-phosphide reaction layer in the phosphorus systems on steels. Lowering the brazing temperature with phosphorus (C1) is paid for by slower removal of the depressant and a narrower maximum brazing clearance, so that for the phosphorus grades the clearance rather than the hold is the control parameter. Raising the chromium content improves corrosion resistance but narrows the amorphization window and, above about 10 wt.%, raises the liquidus. The best compositions found so far (for example Ni–20Cr–7.5Si–4.5Fe–1.5B [31]) are therefore compromises rather than extremes. The iron-based ribbons inherit the same trade-offs at a higher temperature level and are, for now, a niche rather than a replacement for the nickel family.

## 8. Comparative Analysis of the Families

The summary characteristics of the amorphous ribbon brazing filler metal families are collected in Table 6, the criterion-by-criterion assessment of the families against C1–C5 is given in Table 7, the brazing-temperature vs. base-material map is given in Figure 8, and the principal axes of comparison are formulated below, drawing in part on general reviews of brazing [17].

In terms of brazing temperature, the families line up in a sequence from the copper–phosphorus ones (650–700 °C) through the zirconium (740–955 °C) and titanium (790–1050 °C, where the lower bound corresponds to the beryllium branch) to the nickel families (900–1200 °C), jointly spanning the practical range of high-temperature brazing. The ranking by glass-forming ability, as far as the reviewed data allow one to be drawn, is different: the nickel metalloid systems amorphize into ribbon up to 70 μm thick and the multicomponent Ti–Zr–Cu–Ni alloys up to 60–70 μm, whereas binary Ti–Cu reaches only 35–45 μm, while the beryllium-bearing zirconium compositions share the top of the ranking with the nickel ones. For many of the systems considered, the best glass-forming compositions lie near the low-melting eutectic regions, and departing from them in metalloid, chromium, or depressant content narrows the processing window; the position of the GFA maximum also depends on the competing crystalline phases and on kinetics and need not coincide with the liquidus minimum (Section 2.1).

With respect to base materials, the families are complementary rather than competing. Steels and heat-resistant nickel superalloys remain the established domain of the nickel systems, since titanium and copper–phosphorus ribbons form continuous but phosphide- or intermetallic-embrittled seams on steels and are admissible only for non-critical parts. Titanium alloys are brazed with titanium and zirconium ribbons, and the choice between them is governed by the required temperature (lower for the zirconium ones) and by whether zirconium is admissible in the composition. Nuclear-grade zirconium alloys require Ti-free zirconium compositions, while copper remains the domain of Cu–Ni–Sn–P. Refractory metals (Mo, W, Nb, Ta) form the category in which filler metals of most families have been applied, from the nickel and copper ones at moderate joint service temperatures to the titanium, zirconium, and beryllium-bearing ones at high temperatures. Ceramics and graphite, by contrast, form the most selective category, since wetting of unmetallized ceramics is provided only by the active titanium- and zirconium-bearing compositions.

In terms of TLP-technology maturity, the nickel systems lead with quantitatively studied kinetics, in which for the boron–silicon compositions the rate-limiting role belongs to silicon rather than boron [87,181]. The titanium systems are kinetically faster owing to the high diffusional mobility of Cu and Ni in the β phase [143]; for the zirconium systems TLP is likewise the joint-formation mechanism and similar kinetics are expected by analogy, but they have not been measured systematically (Section 6.1), whereas for the copper–phosphorus systems the TLP kinetics have been studied only fragmentarily. In commercial maturity, judged by the number of suppliers and the breadth of the product range, the ranking is headed by the nickel and copper–phosphorus ribbons, with mass production, the broadest product range, and a well-established casting technology; the titanium ones follow with stable niches, and the zirconium ones close the list with single suppliers and small-batch applications. These rankings are a synthesis of the reviewed literature rather than established metrics: the data behind them come from different laboratories, base materials, and test methods (Table 4 and Table 6), and they should be read as orderings rather than as quantitative comparisons.

To close the comparison, let us establish the place of amorphous ribbons within the industrial filler-metal classifications whose groups were listed in the Introduction. What matters is how the AWS A5.8 [15] and ISO 17672 [16] standards relate to the subject of the present review.

Both classifications group filler metals by base and nominal composition and distinguish neither the alloy’s ability to amorphize nor the product form. The most recent editions have not changed this: ISO 17672:2024 [16] added four silver and two Ni–Cr–P–Si compositions to the existing seven classes, and AWS A5.8M/A5.8:2026 [15] retains the same grouping by base. Within the single BNi group, the compositions BNi-1, BNi-2, BNi-3, BNi-9, BNi-5a, BNi-6, BNi-7, and BNi-12 are produced as amorphous ribbon, whereas BNi-5, BNi-10, and BNi-11 are, to our knowledge, available only as powder, and the AWS code carries no trace of this distinction.

Moreover, entire families considered in the present review remain outside the classification. No AWS code exists for the whole zirconium family (Zr–Ti–Cu–Ni, Zr–Cu–Fe–Be), for the iron-based and beryllium-bearing (Ti–Zr–Be, Ni–Si–Be) filler metals, or for the amorphous copper–phosphorus Cu–Ni–Sn–P ribbons, since the BCuP group (the CuP group in ISO 17672 [16]) covers only the nickel-free Cu–P, Ag–Cu–P, and Cu–Sn–P alloys. Of the twelve systems in the summary Table 6, only five carry a classification. In Figure 8 and Table 6, the unclassified entries are marked with §.

Fundamentally, the attribute “classified in AWS” and the attribute “amorphizes into ribbon” are independent, as demonstrated by the matrix in Table 8, all four cells of which are populated. Classified systems that are not produced as amorphous ribbon include the powder grades BNi-5 (which does not amorphize) and BNi-10 and BNi-11 (no amorphous ribbon reported), and the rolled silver BAg and aluminum BAlSi filler metals. Unclassified amorphous ribbons include Cu–Ni–Sn–P and the zirconium and iron systems. The reason is that the standards grew up around traditional crystalline filler metals and contain no attribute for the very property that constitutes the subject of the present review. This is precisely why amorphous ribbon brazing filler metals have to be described cross-system, through the review’s own framework of criteria C1–C5, rather than through the existing classification.

## 9. Conclusions

1. Of the hundreds of systems that amorphize on quenching from the melt, four families have become established as industrial ribbon brazing filler metals: Cu–Ni–Sn–P; the titanium-based Ti–(Zr)–Cu–Ni; the zirconium-based Zr–Ti–Cu–Ni and Zr–Cu–Fe–Be; and the nickel-based Ni–Cr–Si–B and Ni–Cr–P. Together they span the practical range of high-temperature brazing from 650 to 1200 °C and are produced as ribbons 20–80 μm thick at cooling rates of 10^5^–10^6^ K/s. This selection is not accidental. The class exists because, in deep eutectics, the functions of a low-melting depressant and a glass former coincide, and an industrial niche is won only by systems that simultaneously satisfy criteria C1–C5.
2. Criteria C1–C5 form a unified analytical framework for comparing the families (Table 7, Figure 8). They explain both the niche of each family and the reasons why certain compositions remain outside mass production, namely the beryllium-containing ones (C5: special handling, small-batch production), Al–Si (no amorphous phase forms in the binary system, C2), and Fe–Si–B (the brazing temperature for steels is too high, C1). The framework is also consistent with the newest developments. By criteria C1–C5, phosphorus-containing iron-based systems of the Fe–Cr–Ni–P–Si type are expected to form a family of amorphous ribbons for brazing steels; the commercial Fe–Cr–Ni–P–Si powders F300/F302 [13,14] and the first research-scale amorphous iron-based brazing foil [189] are consistent with this expectation, although the evidence is still limited to a few compositions and in industrial maturity the family still lags far behind the nickel-based one (Section 7.7).
3. TLP bonding is the mechanism common to the three families used on metallic base materials (titanium, zirconium, and nickel; Section 2.4), and a thin homogeneous ribbon is a carrier particularly well suited to it. For the titanium-based systems, TLP has been shown to remove the intermetallics from the seam, with joint strength reaching the level of the base metal (about 750 MPa for OT4 and 905 MPa for VT6-s brazed with STEMET 1202 [141]); for the zirconium family the same mechanism is expected, but its kinetics have not been measured systematically (Section 6.1). For the nickel-based systems, the kinetics are limited by the slow diffusion of silicon, and optimization of the ribbon composition yields up to 500 ± 40 MPa on steels. For phosphorus-containing nickel-based ribbons, isothermal solidification on nickel bases is achievable in principle and obeys the classical square-root-of-time law [109], but proceeds slowly, and the brazing clearance remains the practical control parameter. In brazing with copper–phosphorus filler metals, TLP is hardly used at all; in our interpretation, short production cycles and a residual eutectic that is acceptable for heat-exchanger applications make prolonged isothermal solidification economically redundant (Section 3.3).
4. The families are largely complementary rather than competing with respect to the base materials they braze. Steels and heat-resistant alloys remain the domain of the nickel-based filler metals; titanium, of the titanium- and zirconium-based ones; the zirconium alloys of nuclear engineering, of the titanium-free zirconium-based compositions; and copper and heat-exchange equipment, of the copper–phosphorus ones. Within the class of amorphous ribbons, ceramics and graphite are wetted only by the active titanium- and zirconium-containing compositions, whereas refractory metals have been brazed with filler metals of most of the families.
5. The ductile silver- and aluminum-based filler metals delineate the boundary of the class as a “negative control”. Wherever an alloy can be rolled into foil without rapid quenching, or where an amorphous phase does not form in principle, the amorphous ribbon form offers no advantage and is not used.
6. The further development of the class is tied to replacing expensive and toxic components and to moving toward computation-based assignment of brazing schedules. The most obvious directions include the search for beryllium-free low-melting zirconium-based eutectics, iron-based ribbons as an economical replacement for nickel-based ones, and the transfer of quantitative models of isothermal solidification from the nickel-based systems to the titanium-based and copper–phosphorus ones.
7. Of the statements above, the first, fourth, and fifth summarize results demonstrated experimentally in the reviewed literature. The second and third contain the authors’ interpretation: the C1–C5 framework with its operational thresholds, the reading of TLP as the mechanism common to the families used on metallic base materials, and the structural-heredity hypothesis of Section 2.1, which we regard as unproven. The sixth lists directions for future work.

## Figures and Tables

**Figure 1:**
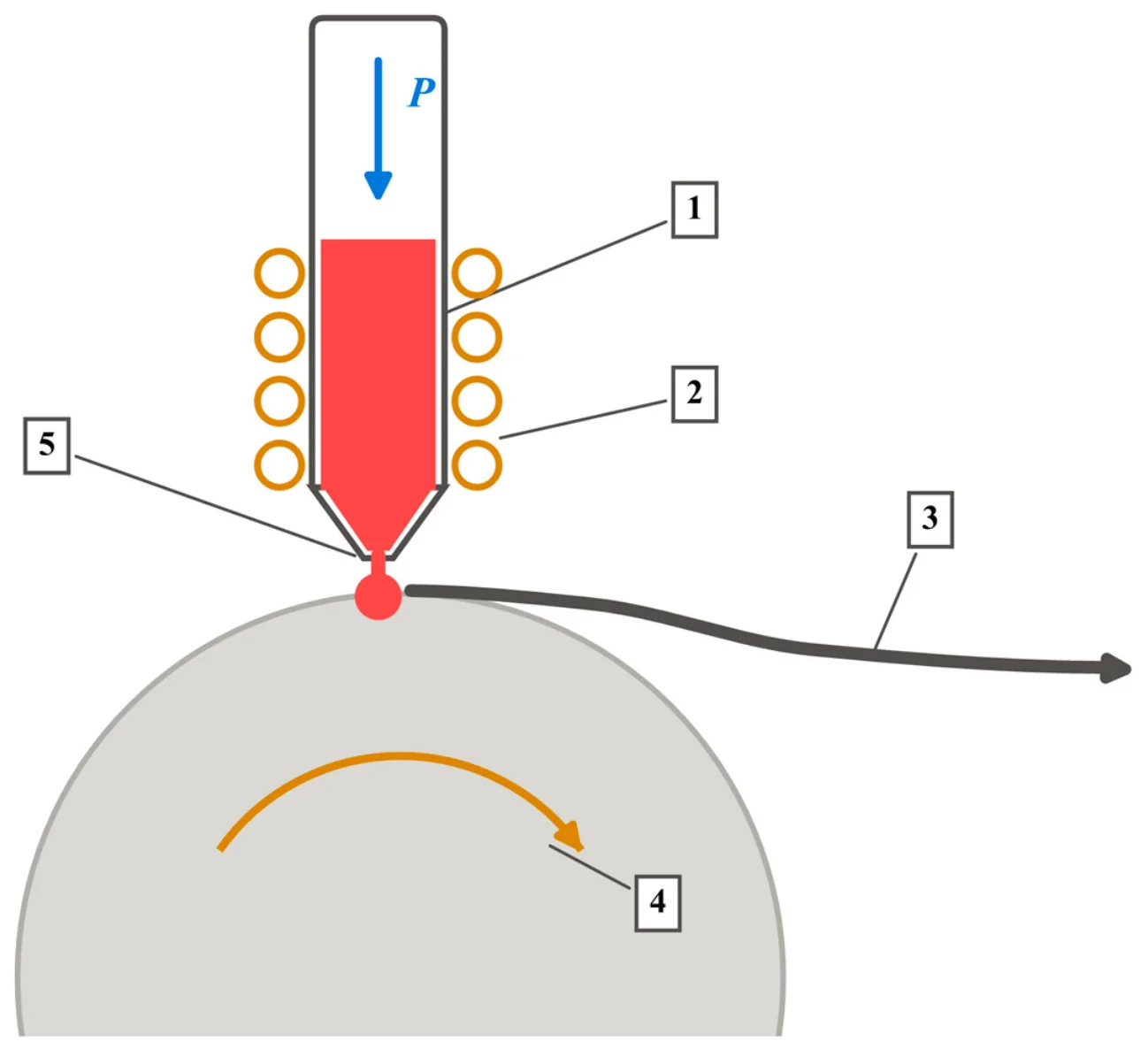
Schematic diagram of single-wheel melt spinning. 1: Crucible containing the melt, from which a jet of melt issues through a nozzle under an excess pressure P; 2: induction coil heating the crucible; 3: finished amorphous ribbon leaving the wheel; 4: rotating cooled quenching wheel (copper, copper–beryllium, or steel) providing a melt cooling rate of ~10^5^–10^6^ K/s; 5: melt.

**Figure 2:**
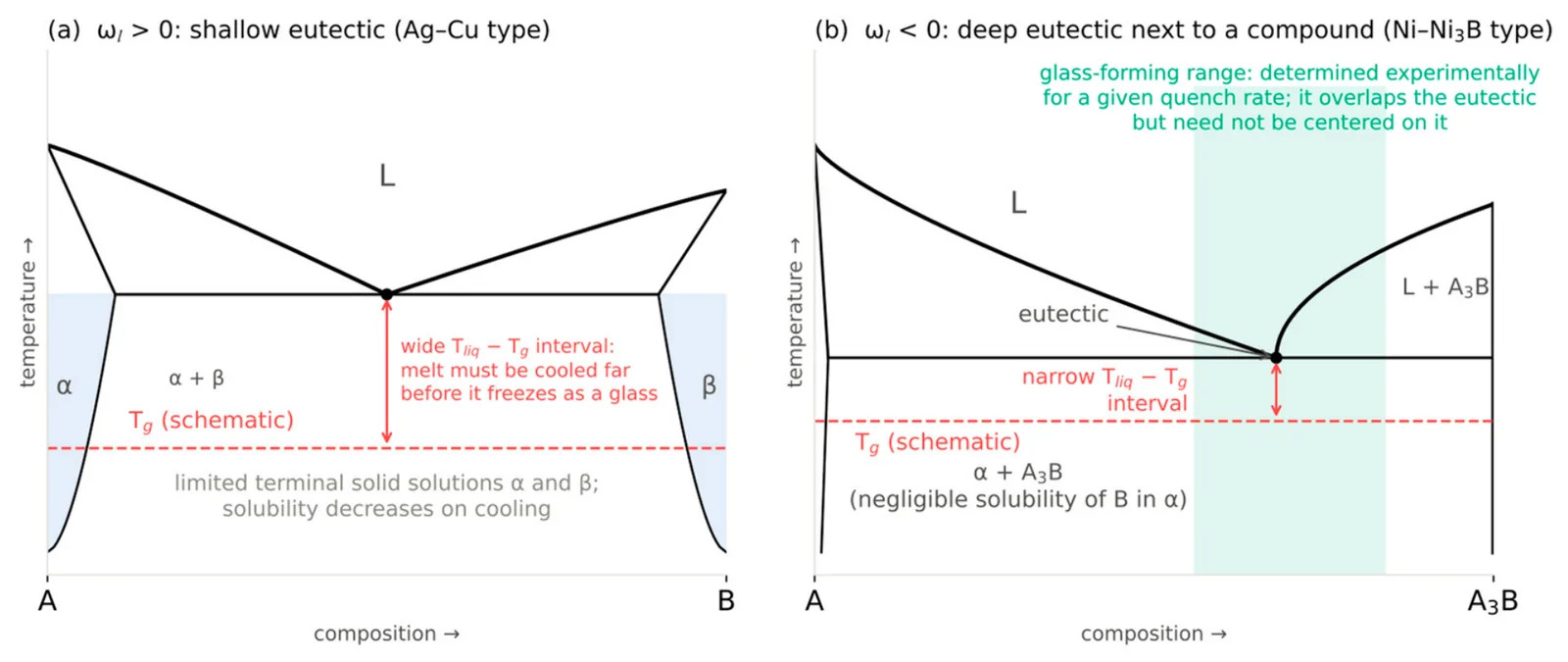
Two characteristic kinds of eutectic phase diagram, drawn schematically and not to scale. (**a**) Positive deviation from ideality in the melt (ω_l_ > 0, Ag–Cu type): a shallow eutectic between limited terminal solid solutions whose solubility decreases on cooling. (**b**) Negative deviation (ω_l_ < 0, Ni–Ni_3_B type): a deep eutectic adjacent to an intermediate compound, with negligible terminal solubility. The panel ends at the compound. The subscript l indicates that ω refers to the melt. The dashed lines mark the glass transition temperature T_g_ schematically, and the arrows mark the interval T_liq_ − T_g_ that the melt has to pass on cooling. A shallow eutectic leaves this interval wide and a deep eutectic narrows it, which is one reason why the depression of the liquidus favors glass formation. The shaded band in (**b**) is the glass-forming range, drawn schematically: it illustrates a possible experimentally determined range for a given quench rate and does not refer to a specific composition, and it overlaps the eutectic but need not be centered on it. Whether a given melt actually vitrifies on melt spinning depends on the quenching conditions and has to be established for each composition (Table 1).

**Figure 3:**
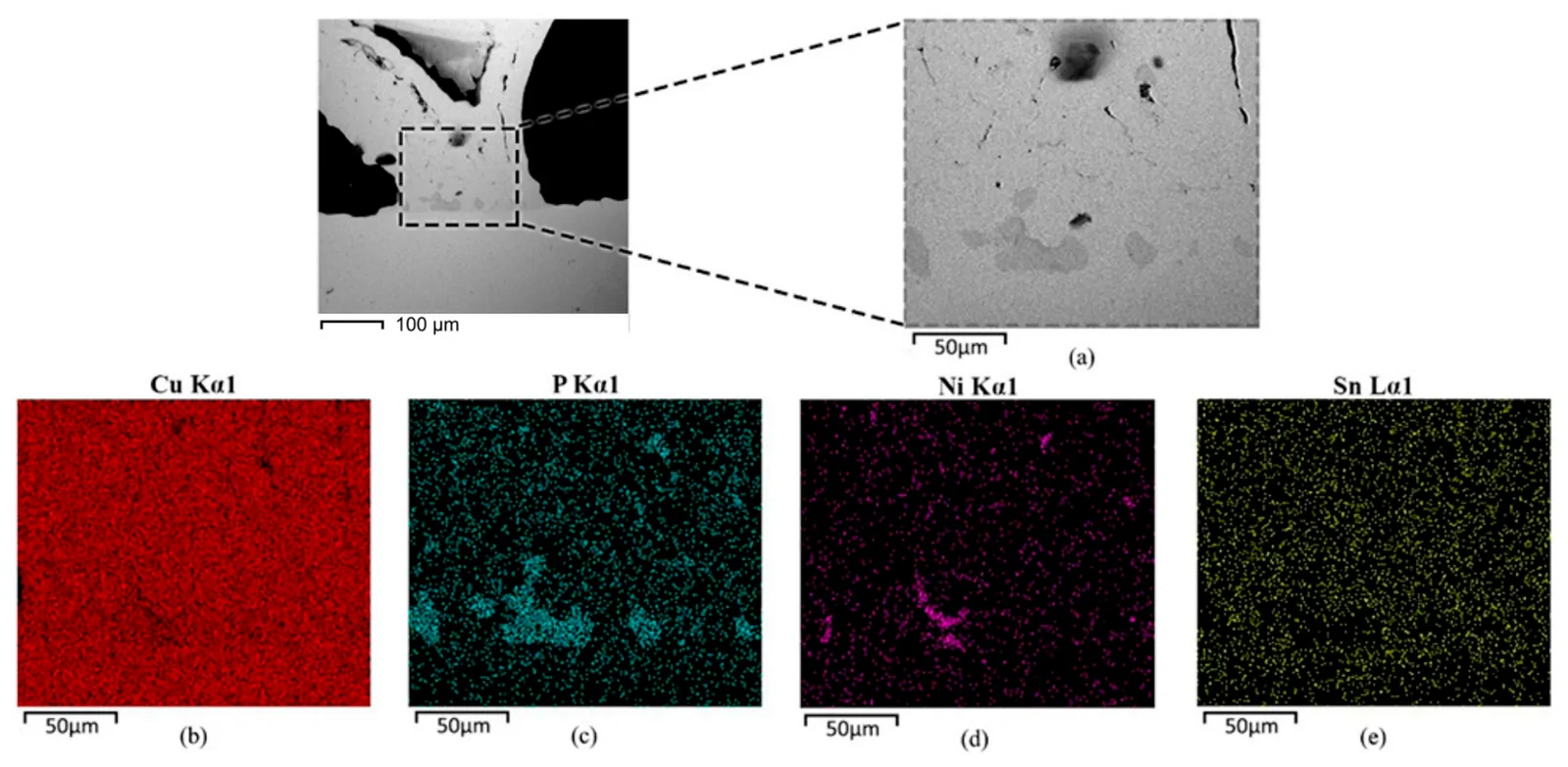
Copper joint brazed with a Cu–Ni–Sn–P amorphous filler metal at 680 °C for 10 min (porous copper joined to a copper plate): (**a**) SEM micrograph of the joint interface on the base side; EDS maps of (**b**) Cu, (**c**) P, (**d**) Ni, and (**e**) Sn. The seam consists of a copper solid solution and a continuous phosphorus-rich reaction product with nickel-enriched regions; phosphorus and nickel remain concentrated in the seam. Adapted from [116] (CC BY 4.0); the instrument footer and scale-bar label have been reformatted for readability.

**Figure 4:**
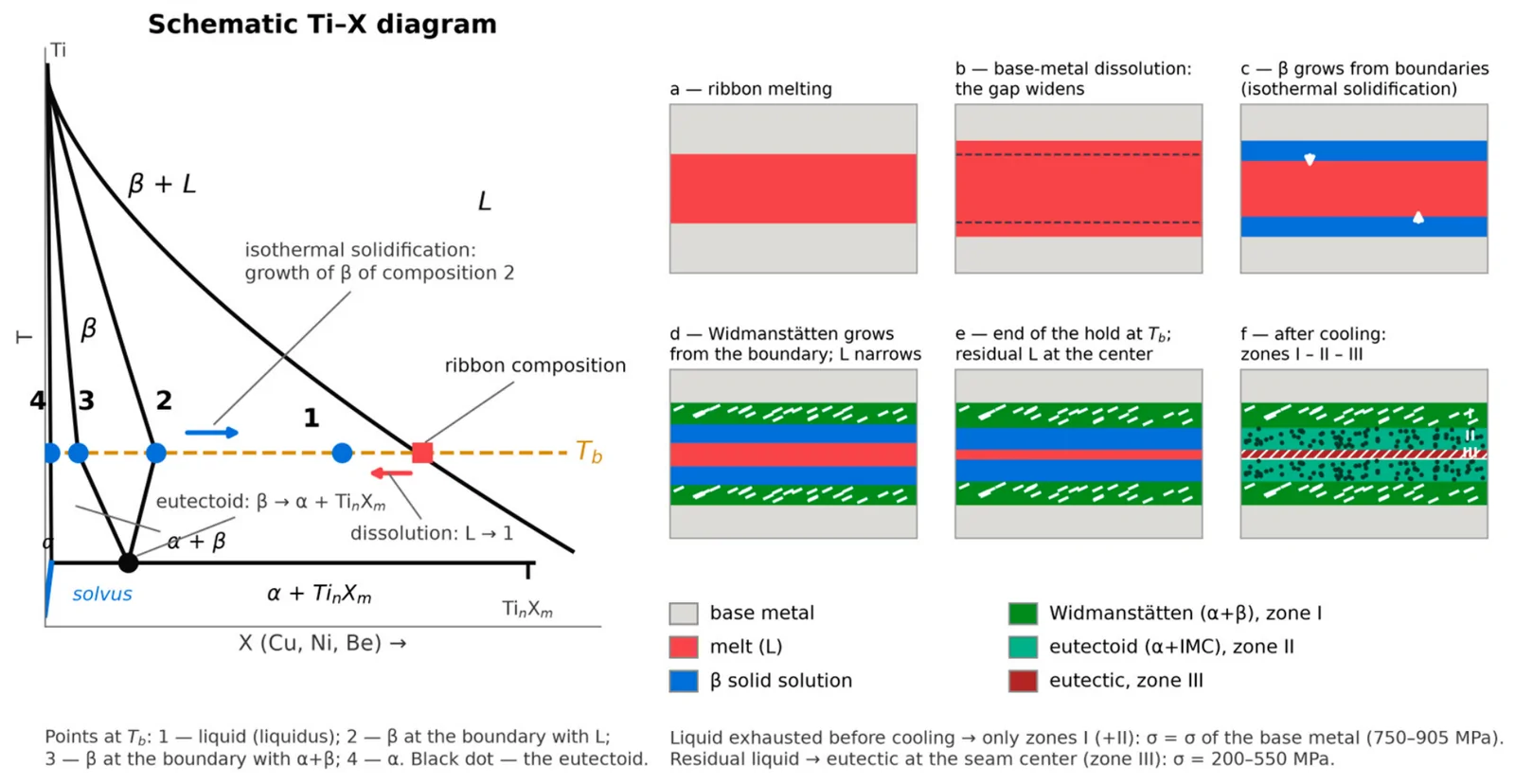
Schematic Ti–X phase diagram, where X denotes the total depressant content (Cu + Ni, plus Be where present), on the left, and the stages of seam evolution, on the right, after [141]. The diagram is schematic: the concentration axes do not convey quantitative compositions and serve only to illustrate the sequence of phase transformations. The case shown is one in which isothermal solidification is incomplete.

**Figure 5:**
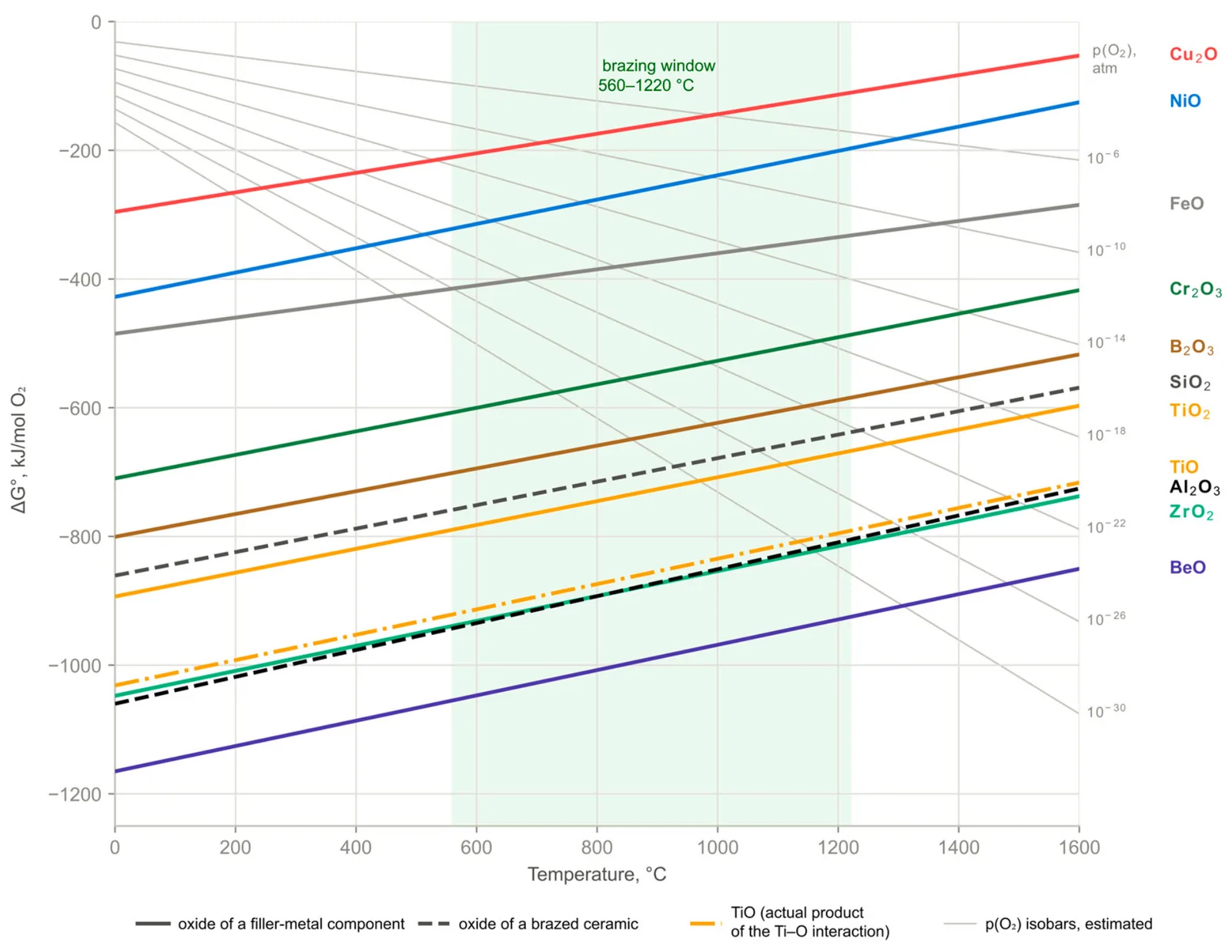
Ellingham diagram for the oxides involved in active brazing. Shown is the standard Gibbs energy of oxide formation, referred to 1 mol O_2_, in the linear approximation ΔG° = ΔH°_298_ − T·ΔS°_298_ from NIST-JANAF data [145]. Solid lines correspond to the oxides of filler-metal components, dashed lines to the oxides of the ceramics being brazed; the dash-dotted TiO line shows the suboxide reported as the interaction product in the alumina-brazing studies cited in the text; the ZrO_2_ line corresponds simultaneously to a filler-metal component and to a ceramic. Thin gray rays show the oxygen partial-pressure isobars; for a real vacuum furnace they give only an estimate, since the oxygen potential of the atmosphere is set by the H_2_/H_2_O and CO/CO_2_ ratios rather than by the total residual pressure. The shaded band marks the brazing-temperature range of the families under consideration (560–1220 °C). The diagram is constructed for pure condensed phases at unit activity; in the filler-metal melt the activities of titanium and of the reaction products are below unity, which shifts the equilibria (see text).

**Figure 6:**
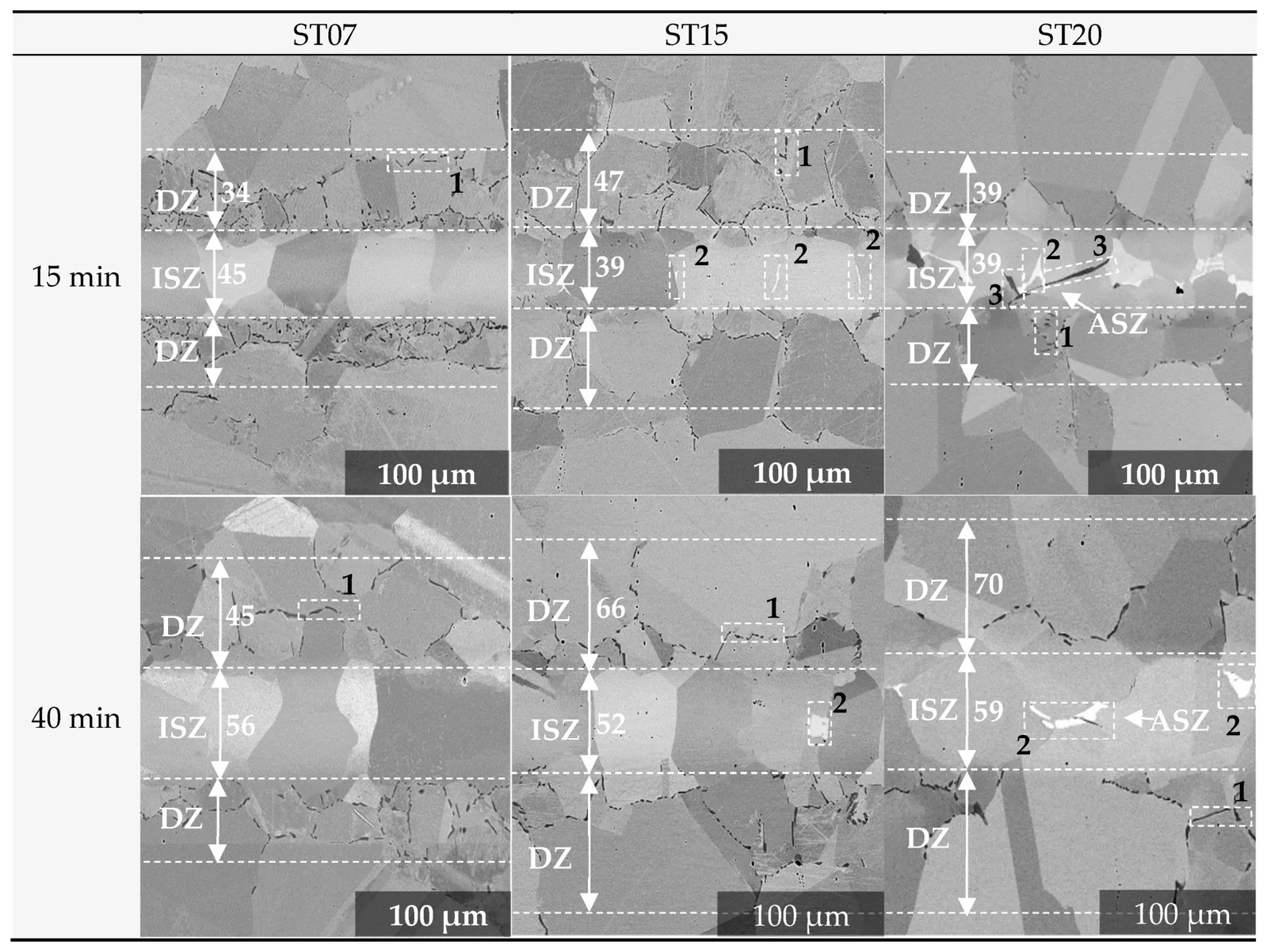
Seams of AISI 304L steel brazed in vacuum at 1160 °C for 15 and 40 min with 45 μm amorphous Ni–Cr–Si–B foils containing 7 (ST07), 15 (ST15), and 20 wt.% Cr (ST20); SEM, backscattered electrons. Dashed lines delimit the zones, white numbers are their widths in μm: ISZ, isothermally solidified nickel solid solution; DZ, diffusion zone of the steel with fine chromium borides on the grain boundaries (1); ASZ, residual liquid solidified on cooling into silicides (light, 2) and borides (dark, 3). More chromium keeps silicides and borides at the seam center; a longer hold widens the ISZ and coarsens the borides in the DZ. Reproduced from [171] (CC BY 4.0).

**Figure 7:**
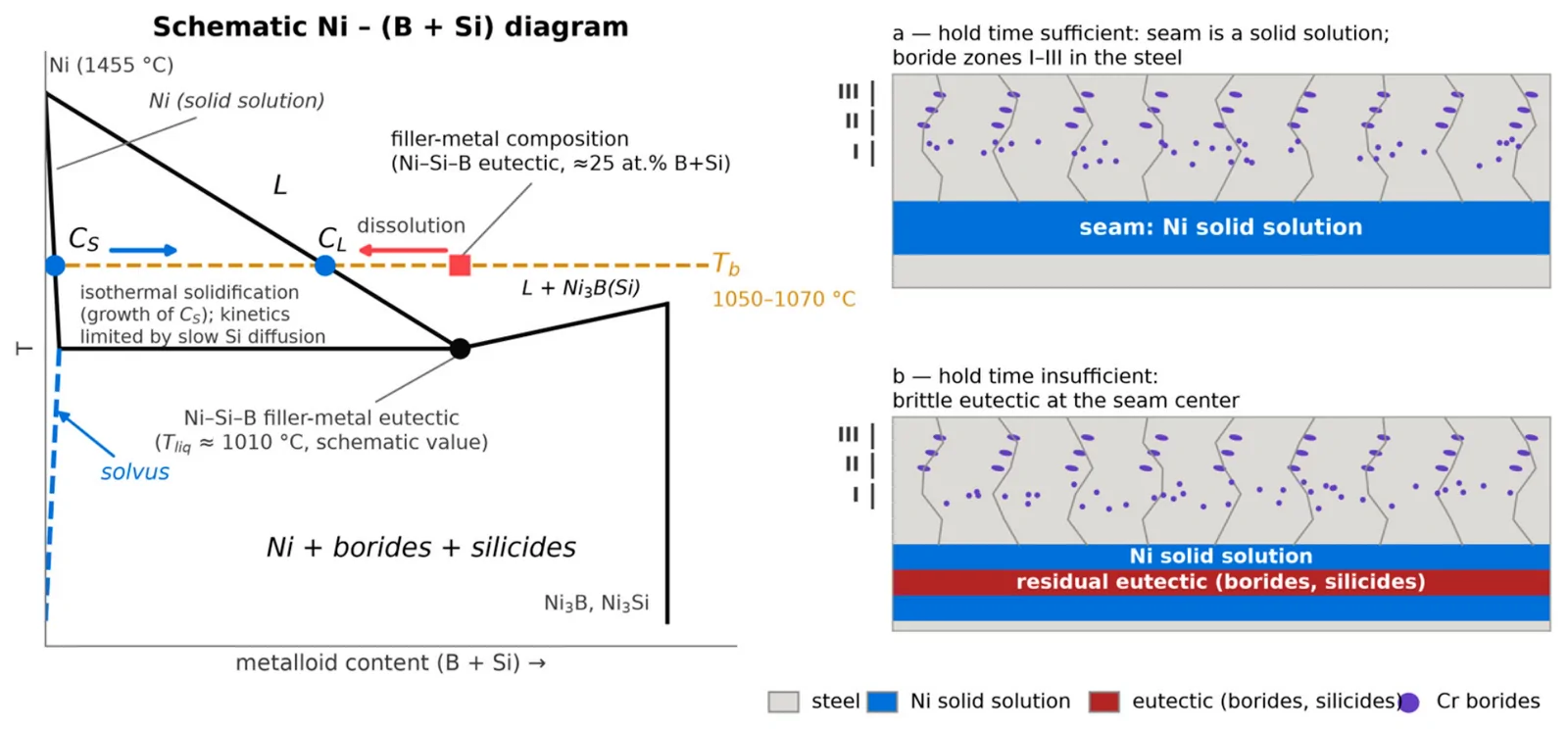
Schematic Ni–(B + Si) diagram with the points C_S_ and C_L_ (**left**) and the two outcomes of the isothermal hold in steel brazing (**right**), after [31]. The temperature scale is schematic: the liquidus marked as ≈1010 °C in the figure is a rounded schematic value, whereas the catalog liquidus of STEMET 1301 is 1020 °C [53] and that of BNi-2 is 1024 °C [15]; the difference does not affect the sequence of stages described. The boride zones in the steel are arranged with increasing distance from the seam: zone I contains fine Cr borides inside the grains, in zone II borides cover most of the grain boundaries, and in zone III borides remain only on isolated boundary segments.

**Figure 8:**
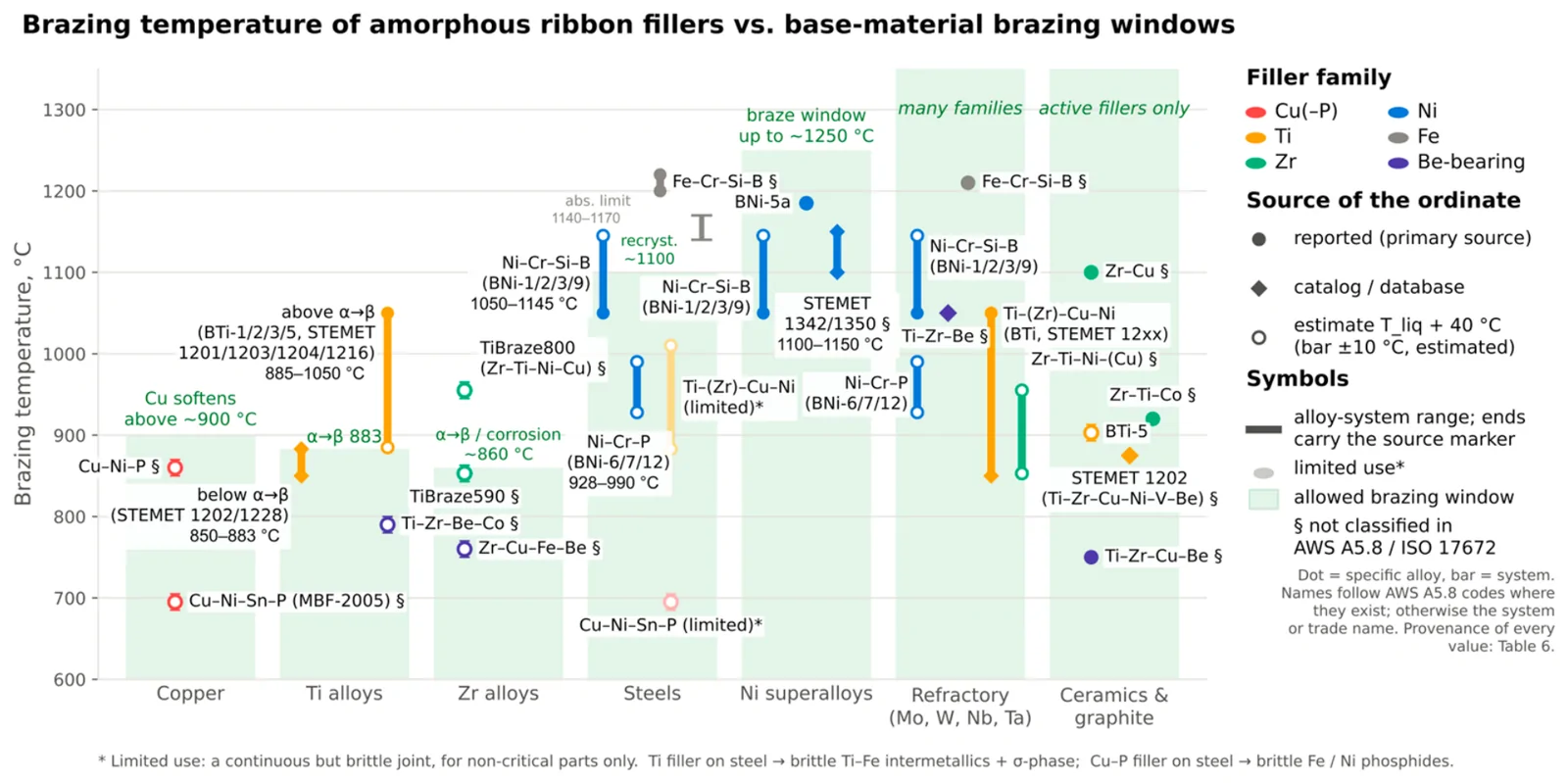
The brazing-temperature vs. base-material map for amorphous ribbon brazing filler metals. Each point corresponds to a specific alloy, each vertical interval to a system. Alloys are named by their AWS A5.8 [15] code or, where no code exists, by alloying system or trade name. The § symbol marks compositions without an AWS A5.8/ISO 17672 [15,16] classification; pale shading indicates limited applicability (a continuous but brittle seam). The ordinate corresponds to the brazing temperature, which typically exceeds the liquidus temperature of the filler metal by 30–50 °C; exotic cases of sub-liquidus brazing are not considered in this review. The source of every ordinate is coded by the marker. Filled circles are brazing temperatures reported in the primary sources, diamonds are values from the manufacturers’ catalogs or databases, and open circles are estimates T_liq_ + 40 °C (the typical superheat being 30–50 °C), shown with a bar of ±10 °C, which is the range implied by the 30–50 °C superheat and not a measured uncertainty. The ends of the system bars carry the same markers, and the provenance of each value (E, C, or L) is marked in the T_braze_ column of Table 6. The STEMET grades are listed in the manufacturer’s catalog [53] and in registered technical specifications; independent sales data are not available to us, and commercial status is not used in ranking the families. Green bands show the admissible brazing-temperature windows of the base materials. For steels and heat-resistant nickel superalloys the windows are shown separately, and the titanium compositions are grouped into intervals below and above the α → β transformation (883 °C). The STEMET 1202/1228 interval is shown only up to the α → β transformation (883 °C); the catalog window of these grades extends to 900 °C.

**Table 2:** Operational thresholds used in this review for applying criteria C1–C5 (proposed by the authors).

Criterion	Quantity Assessed	Met (✓)	Met with Reservations (△)	Not Met (✗)	How Determined
**C1 Brazing temperature**	Brazing temperature T_braze_ = T_liq_ + 30–50 °C against the limit T_lim_ of the base material: most industrial steels 1100 °C (onset of recrystallization and grain growth; 1140–1170 °C absolute) [70,73]; heat- resistant Ni alloys ≈ 1250 °C [51]; unalloyed Ti 883 °C and Zr 863 °C (α → β) [53], for an alloy its own transus; Cu ≈ 1000 °C	T_braze_ ≤ T_lim_ − 30 °C over the whole brazing window of the grade	T_lim_ − 30 °C < T_braze_ ≤ T_lim_, or only part of the brazing window of the grade lies at or below T_lim_ − 30 °C	T_braze_ > T_lim_	DSC/DTA liquidus (10–20 K/min); catalog brazing window
**C2 Glass formation**	Critical thickness t_cr_ of a fully X-ray-amorphous ribbon at the cooling rate of industrial melt spinning (10^5^–10^6^ K/s [25])	t_cr_ ≥ 40 μm	20 μm ≤ t_cr_ < 40 μm, or ribbon reported only as amorphous–crystalline without a fully amorphous t_cr_	t_cr_ < 20 μm, or no amorphous phase forms	XRD (absence of Bragg peaks); DSC (crystallization exotherm; a resolved glass transition is not required); TEM where required
**C3 Base-material compatibility**	(a) Joint strength after the optimum schedule relative to the base metal, σjoint/σbase, from the same type of test for joint and base metal (the governing quantity); (b) controllability of the brittle phases; (c) wetting without flux, contact angle θ, used only where strength data are absent	σjoint/σbase ≥ 0.7, with brittle phases removable by TLP or confined to an acceptable fraction; where strength data are absent, θ ≤ 30°	0.4 ≤ σjoint/σbase < 0.7 (continuous but embrittled seam; “limited applicability” in Section 8); where strength data are absent, θ ≤ 60° (wetting alone does not establish strength)	σjoint/σbase < 0.4, an unavoidable continuous brittle layer, or no wetting	Sessile-drop test [81]; tensile or shear tests at room temperature; SEM/EDS of the seam
**C4 Ribbon processability**	(a) Ductility of the as-cast ribbon in bend tests: the tight 180° bend (bend radius equal to the ribbon thickness) and the number of reverse 90° bends to fracture according to GOST 13813-68 [82,83]; (b) industrial casting width; (c) retention of ductility on storage, noted where data exist but not graded, since one-year data are not available for most grades	Tight 180° bend without fracture; width ≥ 50 mm	Fails the tight 180° bend but withstands at least 10 reverse 90° bends, which is sufficient for coiling and for stamping simple preforms; or width from 20 mm to below 50 mm (narrow or single-supplier casting)	Fewer than 10 reverse bends (fracture at the first bends or on coiling); width < 20 mm	Tight-bend and reverse-bend tests (manufacturers’ quality control [53,60]; GOST 13813-68); catalog widths
**C5 Cost and safety**	(a) Presence of toxic or regulated elements (Be; Cd and Pb, restricted under REACH); (b) indicative raw-material cost of the alloy components, in USD per kg of alloy at 2024–2026 exchange prices	No toxic or restricted elements; component cost below about 100 USD/kg (Cu, Ni, Cr, Fe, Ti, Zr, Si, B, P, Sn)	Beryllium-bearing compositions (special handling in ribbon production and brazing), or component cost of about 100–1000 USD/kg (Ag-, Ge-, and rare-earth-metal-bearing compositions)	Cd-containing (restricted under REACH), or precious-metal-based (Pd, Pt, Au) with component cost above about 1000 USD/kg	REACH Annex XVII; IARC classification; exchange prices of the metals (indicative; the ranking rather than the absolute values is used)

Note: The thresholds are an operational proposal of the authors for the comparison in this review and are not part of any standard. T_liq_ denotes the liquidus temperature of the filler metal, t_cr_ the critical thickness of fully amorphous ribbon, θ the contact angle on the unfluxed base material, and σ_joint_/σ_base_ the ratio of the joint strength to that of the base metal under the optimum brazing schedule. The cost bands are indicative order-of-magnitude values of the raw-material cost of the components at 2024–2026 prices; it is the ranking rather than the absolute values that is used. The reverse-bend test of GOST 13813-68 consists of repeated 90° bending of the ribbon to alternate sides about a mandrel until fracture, the result being the number of bends withstood. Where the data needed for a criterion are not reported, the assessment in Section 8 is “not assessed” rather than ✓; where the grades of one family fall into different categories, they are listed individually. Bold type identifies the criterion labels (C1–C5).

**Table 3:** Independence of glass-forming ability (C2) and industrial ribbon processability (C4): the systems of this review in the four combinations of the two criteria.

	C4 Met: Ductile Ribbon of Industrial Width Is Obtained	C4 Not Met or Met with Reservations
**C2 met (fully amorphous ribbon at industrial quenching rates)**	Ni–Cr–Si–B and Ni–Cr–P (BNi-2, BNi-7, STEMET 1301; ribbon up to 70 μm, widths up to 200 mm [53,60]); Ti–Zr–Cu–Ni (BTi-3, MBF-5000 series, STEMET 12xx [19,53]); Cu–Ni–Sn–P (MBF-2005, STEMET 1101 [53,60]). The industrial core of the class (Section 3, Section 5 and Section 7).	Zr-based ribbons: the reactive melt wets the casting nozzle and oxidizes at the wheel, so calibrated ribbon is usually cast 20–30 mm wide, and wider ribbon, although feasible, demands much greater effort [74,75] (Section 6.4); phosphorus-rich Ni–P glasses [78] and Fe–B–Si–C-type glasses [77], which embrittle on structural relaxation well before crystallization (Section 2.2, C4).
**C2 not met (no fully amorphous ribbon at industrial quenching rates)**	Ni–Si–Be: ductile nanocrystalline ribbon (Ni–5Si–3Be) [79,80] (Section 7.4); Al–Ge–Si: microcrystalline ribbon for steel/aluminum joints [83,84,85,86] and cast Al–Si eutectic ribbon [84,85] (Section 4.1); partially amorphous Ag–Cu–In foil (US4182628) (Section 4.2). Ribbon without vitrification; outside the formal scope (Section Scope and Literature Search Methodology).	BNi-5 (Ni–19Cr–10Si), BNi-10 and BNi-11, VPr24/32/36/37: silicon-only or low-metalloid compositions produced as powder and paste only (Section 7.4 and Section 8).

Note: Bold type identifies the row categories according to criterion C2.

**Table 6:** Summary characteristics of the families of amorphous ribbon brazing filler metals.

System/Family AWS A5.8/ISO 17672 [15,16]	Grade	Composition, wt.% (at.%)	T_sol_–T_liq_, °C	T_braze_, °C (E/C/L, See Note)	GFA, Critical Ribbon Thickness	Base Materials, Applications, Status
Cu–Ni–(Sn)–P — *	Metglas MBF-2005/MBF-2007	Cu–5.7Ni–9.6Sn–6.5P (76Cu–6Ni–5Sn–13P at.%)	T_liq_ 655	~695 ^L^	P is the classical metalloid glass former. Standard ribbons 25–50 μm (Metglas).	Copper and its alloys; heat exchangers, HVAC (heating, ventilation, and air conditioning), refrigeration equipment. Self-fluxing on copper. STEMET 1101: brazing of Be/CuCrZr in the ITER first wall. On steels limited use: Fe phosphides embrittle the seam, non-critical parts only. Limitations: corrosion (seawater, S), brittleness. Status: commercial.
	OKC600	Cu–4.2Ni–15.6Sn–5.3P	T_liq_ 610	~650 ^L^		
	STEMET 1101	Cu–8.8Ni–3.5Sn–7.0P	615–650	650–700 ^C^		
	STEMET 1105	Cu–5.4Ni–9.2Sn–6.3P	615–650	650–700 ^C^		
	Russia (crystalline): PMF, PMFOTsr	Cu–(7–30)Ni–(15–20)P (at.%, patent compositions)	670–820	—		
Ti–Cu (binary) —	STEMET 1203	Ti–50Cu	≈980	1000–1050 ^C^	Ti50Cu50 is the best binary glass former (the [Ti9Cu6]Cu3 cluster), but its GFA is moderate (no glass transition resolved [126]). Critical thickness 35–45 μm [52,76].	Titanium and its alloys, copper, ceramics (metal–ceramic joints), refractory metals (Mo, W, Nb, Ta); thin-walled structures. Status: commercial.
	STEMET 1204	Ti–72Cu	≈875	950–1000 ^C^		
Ti–Cu–Ni BTi-1, BTi-2	BTi-1 (Ticuni, Ti-flo 950, ANA 3, Meta-Braze BTi-1)	Ti–15Cu–15Ni	910–960	970–1050 ^C^	Glass formation poorer than in Ti–Zr–Cu–Ni; amorphous ribbon is feasible.	Titanium alloys (VT1-0, VT6/TC4), ceramics, refractory metals; honeycomb structures, heat exchangers. Status: commercial (the series-produced Russian ribbon belongs to the Ti–Zr–Cu–Ni family).
	BTi-2 (Ticuni-60, Meta-Braze BTi-2)	Ti–15Cu–25Ni	890–940	~980 ^L^		
Ti–Zr–Cu–Ni (+ Ag, Sn, Co, Fe, V, Pd, Be) BTi-3, BTi-5	BTi-3 (MBF-5002, TiBraze375, Meta-Braze BTi-3)	Ti–37.5Zr–15Cu–10Ni	825–843	~885 ^L^	60–70 μm is the working range [53,76]. Amorphicity is retained with Cu + Ni down to 12 at.% when Sn/Co/Fe/Al are added [72]. First ribbons: [19], width up to 12.5 mm.	Ti alloys, TiAl, Ti_2_AlNb, refractory metals (Mo, W, Nb, Ta), dissimilar pairs (TC4/SS316L, TC4/IN718). Ceramics: oxide ceramics (STEMET 1202 wets them actively → metal–ceramic joints), ZrO_2_/29NK, ZTA (endoprostheses). Zr strengthens the solid solution; Cu and Ni embrittle it (Ti_2_Cu, Ti_2_Ni). Status: commercial, the primary choice for titanium.
	BTi-5 (TiBraze200, Meta-Braze BTi-5)	Ti–20Zr–20Cu–20Ni	848–863	~905 ^L^		
	STEMET 1201	Ti–12Zr–24Cu–12Ni	820–865	900–1000 ^C^		
	STEMET 1202	Ti–12Zr–22Cu–12Ni–0.8V–1.5Be	750–815	850–900 ^C^		
	STEMET 1216 (VPr16)	Ti–12.5Zr–22.5Cu–9Ni	875–900	950–1000 ^C^		
	STEMET 1228 (VPr28)	Ti–23Zr–17Cu–15Ni	810–850	850–900 ^C^		
Ti–Zr–Be (+ Nb, V, Co) —	Ti–48Zr–4Be	Ti–48Zr–4Be	—	1050 ^E^	At Be > 3%, amorphous/nanocrystalline foils are obtained by melt spinning. Liquidus lowered by ≈150 °C per 1% Be.	Refractory metals (Mo, Nb, W, Ta), graphite, SiC, Cf/SiC composites, ceramics. Nuclear engineering (DEMO: 48Ti–48Zr–4Be). Problem: brittle beryllides (Ta_2_Be, TiBe). Status: niche (nuclear engineering, ceramics).
	Ti–47.4Zr–5.6Be–12.2Co (35Ti–25Zr–30Be–10Co at.%)	Ti–47.4Zr–5.6Be–12.2Co (35Ti–25Zr–30Be–10Co at.%)	750	~790 ^L^		
	Ti–40Zr–8.5Nb–1.5Be	Ti–40Zr–8.5Nb–1.5Be	—	—		
Zr–Ti–Cu–Ni (Be-free) —	Zr–16Ti–17Cu–19Ni	Zr–16Ti–17Cu–19Ni	795–835	~875 ^L^	Working foils 30–40 μm. GFA higher than in the titanium counterparts (the compositions lie below the eutectic).	Zircaloy-4/E110, Ti–Zr pairs, ceramics, graphite. Brazing below the α → β transformation. Limitation: Ti accelerates seam corrosion in reactor water. Status: niche (nuclear engineering).
	TiBraze590	Zr–17.5Ti–21.5Ni	796–813	~850 ^L^		
	TiBraze620	Zr–11Ti–20Ni–7Nb	791–808	~850 ^L^		
	TiBraze800	Zr–15Ti–13Ni–7.5Cu	901–915	~955 ^L^		
Zr–(Ti)–Cu with Be or REM (strong glass formers)	STEMET 1409	Zr–11Ti–12Cu–14Ni–2Nb–1.5REM	690–750	750–850 ^C^	Strong glass formers (REM, Be; the latter enters Vitreloy). Zr–Be binary amorphous ribbons [92].	VVER-440 spacer grids of E110 alloy (with the Ti-free STEMET 1410, Section 6.2), Zr/steel transition joints, Mo/graphite X-ray tube anode. Status: laboratory/small-batch (nuclear engineering).
	STEMET 1410	Zr–8Cu–2Sn–5.5Fe–1Nb–0.4Cr–(2.5–3.5)Be–(0.5–1)Ge (Ti-free; manufacturer’s specification [53])	—	—		
	Zr–(5–20)Ti–(4–7)Fe–(1–3)Cu–(1.5–2.5)Be (patent compositions)	Zr–(5–20)Ti–(4–7)Fe–(1–3)Cu–(1.5–2.5)Be (patent compositions)	690–720	~740 ^E^		
	Zr–22.1Ti–14.1Cu–4.7Be (Zr35–25Ti–12Cu–28Be at.%)	Zr–22.1Ti–14.1Cu–4.7Be (Zr35–25Ti–12Cu–28Be at.%)	673–711	—		
Ni–Cr–Si–B (Ni–Si–B) BNi-1, BNi-2, BNi-3, BNi-9	BNi-1 (MBF-15)	Ni–14Cr–4.5Fe–4.5Si–3B	965–1103	~1145 ^L^	Up to 70 μm at ~3% B (eutectic). Cr at 20% reduces this to 35–45 μm. At 1.2% B/metalloid contents below the eutectic: 30–40 μm.	Stainless steels, nickel heat-resistant superalloys; honeycombs, heat exchangers, aircraft engines, nuclear engineering. Russian counterparts: Asha (75N13KhSR ≈ Ni–13Cr–Si–B; 92NSR ≈ Ni–Si–B, etc.†), VPr11 (Ni–15Cr–4.5Si–2.5B–4Fe). Problem: Cr borides in the seam and DAZ. Status: commercial, mass-produced.
	BNi-2 (MBF-20, STEMET 1301)	Ni–7Cr–3Fe–4.5Si–3.2B	969–1024	1050–1070 ^E^		
	BNi-3 (MBF-30)	Ni–4.5Si–3.2B	984–1054	~1095 ^L^		
	BNi-9	Ni–15Cr–4B	1055 (eut.)	~1095 ^L^		
Ni–Cr–P (Ni–P) BNi-6, BNi-7, BNi-12	BNi-6	Ni–11P	≈880 (eut.)	~920 ^L^	Up to 70 μm at ~10% P (eutectic). General rule for Ni systems: at least 20–25 at.% metalloids.	Steels, thin-walled tubes, honeycombs; nuclear applications (boron-free). Self-fluxing ability, gaps up to 25 μm. Problem: phosphides at the steel interface. Status: commercial.
	BNi-7 (MBF-51)	Ni–14Cr–10P	888 (eut.)	~930 ^L^		
	BNi-12	Ni–25Cr–10P	880–950	~990 ^L^		
Ni–Cr–Si (BNi-5); heat-resistant Ni–Cr–Co–(W, Mo, Nb)–Si–B BNi-5, BNi-5a	BNi-5 (Wall Colmonoy, Höganäs; powders)	Ni–19Cr–10Si	1080–1135	1150–1200 ^C^	BNi-5 does not amorphize: its only metalloid is Si (~10 wt.%), insufficient for glass formation; powder/paste only. BNi-5a (1.5% B) and the low-boron heat-resistant grades STEMET 1342/1350/1309 amorphize into 30–40 μm ribbon.	Steels and heat-resistant alloys under severe corrosive conditions; nuclear applications (boron-free); heat-resistant STEMET 1342/1350 for aircraft engines and thermally stressed assemblies (counterparts of the powder grades VPr42/VPr50). Status: BNi-5 commercial (not as ribbon); STEMET 1342/1350/1309 as ribbon and powder.
	BNi-5a	Ni–19Cr–7.3Si–1.5B	1052–1144	~1185 ^L^		
	STEMET 1342 (counterpart of VPr42)	Ni–14Co–8.5Cr–6W–6.5Si–1.2B	1000–1080	1100–1150 ^C^		
	STEMET 1350 (counterpart of VPr50)	Ni–19Cr–9Co–5Mo–6Si–1.3B–1.2Nb	1020–1120	1140–1150 ^C^		
	STEMET 1309	Ni–15Cr–4Fe–7.5Si–1.5B–4Mo	1044–1130 (DSC)	—		
Ni–(Cr)–Si–Be —	Ni–6Si–5Be	Ni–6Si–5Be	≈915	~955 ^L^	Does not amorphize; at Si < 5 and Be < 3.5%, a ductile nanocrystalline ribbon is obtained.	Austenitic steels (Kh18N10T). Limitation: Be toxicity, oxidation. Status: laboratory (nanocrystalline ribbon).
	Ni–5Si–3Be	Ni–5Si–3Be	≈1130	~1170 ^L^		
Fe–Si–B/Fe–Cr–Si–B/Fe–Cr–Ni–P–Si —	Höganäs F300/F302	F300-10: Fe–20Cr–20Ni–7P–4Si–10Cu [90]; F302: Fe–15Cr–10Ni–9P–5Si–5Cu [14] (gas-atomized powders)	T_liq_ ≈ 1050	1100 [14]; ≥1120 [90] ^E/C^	Fe–Si–B is the base of the Metglas 2605 magnetic ribbons and amorphizes excellently (high GFA), but no commercial filler metal of this composition exists. The actual Fe fillers are the Fe–Cr–Ni–P–Si powders F300/F302 and the experimental Fe–Cr–Si–B amorphous–crystalline ribbon 40–45 μm thick [191]; a research-scale amorphous Fe-based brazing foil has been reported [189]. The brazing temperature exceeds the recrystallization limit of mass-produced steels.	Steel structures, EGR coolers. Motivation: Ni price, environmental concerns. Status: niche, growing interest.
	Experimental Fe–12Cr–Si–B (for EP-823 steel, BREST)	Fe–12Cr–Si–B (Ni-free)	T_m_ 1070–1150	1200–1220 ^E^		
Ag–Cu–(Zn, Sn, In) BAg-1…BAg-37	Cusil and others; PSr (Russia)	Ag–28Cu eutectic; + Zn, Sn, In (Cd excluded under REACH)	600–900	620–900 ^C^	Does NOT amorphize and does not need to: ductile, rolled into foil and wire.	Universal brazing family. Status: negative control (Section 4.2).
Ag–Cu–Ti (active) —	Cusil-ABA, Ticusil (Morgan)	Cusil-ABA: Ag–35.3Cu–1.75Ti; Ticusil: Ag–26.7Cu–4.5Ti	780–900	830–920 ^C^	Does not amorphize; rolled foil, powder, paste.	Active brazing of ceramics, graphite, sapphire. Status: negative control (Section 4.2).
Al–Si (–Cu, –Mg) BAlSi-2…BAlSi-11	Al–7.5Si (BAlSi-2, AA4343)	Al–7.5Si	577–613	600–620 ^C^	No amorphous phase forms (C2); rolled/cast semi-flexible microcrystalline ribbon and rods. Cu lowers T_m_; Mg is an activator (oxide getter) for vacuum brazing.	Aluminum and its alloys, heat exchangers (including vacuum and controlled-atmosphere brazing (CAB)). Status: negative control (Section 4.1).
	Al–12Si (BAlSi-4, AA4047; STEMET 1501)	Al–12Si (577 °C eutectic)	577–582	582–604 (AWS [15]); 560–585 (STEMET 1501 catalog) ^C^		
	Al–10Si–4Cu (BAlSi-3, AA4145)	Al–10Si–4Cu	521–585	570–605 ^C^		
	Al–10Si–1.5Mg (BAlSi-7, AA4004; vacuum)	Al–10Si–1.5Mg	559–596	590–620 ^C^		
Al–REM–TM (Al–Ni–Y, etc.) —	Al–Ni–Y, Al–Y–Ni–Fe, Al–Y–Fe	Al–Ni–Y, Al–Y–Ni–Fe, Al–Y–Fe	above the Al brazing window	—	Amorphize into thin ribbon, but the alloying elements do not act as depressants (C1, C2).	Structural materials (1400–1700 MPa), not filler metals. Status: negative control (Section 4.1).

Note: An “—“ in the classification column means that no AWS A5.8/ISO 17672 [15,16] code exists for the system (in Figure 8 the same entries are marked with §). An “—“ in the temperature and composition columns means that the data are not reported in the primary sources. Superscripts in the T_braze_ column give the source of the brazing temperature: E, reported in the cited experimental study; C, manufacturer’s catalog, data sheet, or standard (AWS A5.8 [15], ISO 17672 [16]); L, estimate T_liq_ + 40 °C (Figure 8). A “~” before a brazing temperature denotes an estimate (T_liq_ + 40 °C, the typical superheat of 30–50 °C above the liquidus); all other values are from the primary sources (for Ti–48Zr–4Be, 1050 is the brazing temperature per the STEMET database). “(eut.)” denotes the eutectic temperature of the system. * The BCuP group (the CuP group in ISO 17672 [16]) covers the nickel-free Cu–P, Ag–Cu–P, and Cu–Sn–P alloys (BCuP-9 = CuP 386); the nickel-bearing Cu–Ni–Sn–P and Cu–Ni–P alloys are not included in it. † Ribbon designations of the Asha Metallurgical Plant: the first number ≈ %Ni, the number before “Kh” ≈ %Cr, “S” stands for silicon and “R” for boron; exact compositions are per the plant’s specifications. REM = rare-earth metals. Temperature ranges of the STEMET grades are per the manufacturer’s official catalog (MIFI-AMETO LLC, stemet.ru); BAlSi temperatures are the standard AWS A5.8 [15] values. Brazing with STEMET grades is carried out in vacuum (≤5·10^−5^–10^−4^ mm Hg) or in pure inert-gas atmospheres; with the copper grades (Cu–Ni–Sn–P), brazing in air with F209 flux is also possible. The bottom four systems (gray shading) are the negative control delineating the boundary of the class (Section 4).

**Table 7:** Application of the selection criteria C1–C5 to the families of amorphous ribbon brazing filler metals, assessed on the representative grades named in Section 2.3 and Table 6, and to the systems that did not pass the selection.

Family	C1 (Brazing T)	C2 (GFA, Ribbon)	C3 (Compatibility with the Base)	C4 (Ribbon Ductility)	C5 (Cost and Safety)
Cu–Ni–Sn–P	✓ 650–700 °C on copper (limit ≈1000 °C)	✓ t_cr_ 50–60 μm (STEMET 1101); △ 20–35 μm for binary Cu–P	✓ copper (joint strength close to that of annealed copper, Section 3.3); △ steels (continuous but embrittled seam: brittle iron phosphides confine use to non-critical parts [17,98], Section 2.3; strength ratio not reported)	✓ ductile ribbon, industrial widths 50–200 mm [53,60]	✓
Ti–Cu–Ni, Ti–Zr–Cu–Ni	△ 850–1050 °C against the 883 °C transus of titanium: ✓ only for the lowest-melting STEMET 1202 brazed at 840–850 °C, △ for the 850–883 °C part of its window, ✗ for BTi-1, BTi-3, and STEMET 1201/1203/1204 (885–1050 °C)	✓ t_cr_ 60–70 μm (Ti–Zr–Cu–Ni); △ 35–45 μm (binary Ti–Cu)	✓ titanium (base-metal strength after TLP [141]) and ceramics (Section 5.5); △ steels (TiFe intermetallics; joint strength 30% or more below that of all-titanium joints [138], Section 5.6)	✓ (MBF-5000 series, TiBraze, STEMET 12xx)	✓ beryllium-free grades; △ STEMET 1202 (1.5 wt.% Be)
Zr–Ti–Cu–Ni	△ 750–955 °C against the 863 °C transus of zirconium: △ STEMET 1409 (750–850 °C; the upper part of the window exceeds 833 °C), △ TiBraze590/620 (≈850 °C), ✗ TiBraze800 (≈955 °C)	△ working foils 30–40 μm [53]; t_cr_ of fully amorphous ribbon not reported	✓ zirconium, titanium; for in-reactor zirconium alloys Ti-free compositions only (Section 6.2)	△ calibrated ribbon usually 20–30 mm wide (Section 6.4)	✓
Zr–Cu–Fe–Be	✓ ≈740 °C (patent compositions); △ up to 850 °C	not assessed: amorphous ribbon reported [92,156], t_cr_ not reported	✓ zirconium, titanium, ceramics	△ same casting constraints as Zr–Ti–Cu–Ni (Section 6.4)	△ beryllium (special handling)
Ni–Cr–Si–B, Ni–Cr–P	✓ on Ni superalloys (900–1200 °C, limit ≈1250 °C); on steels (limit 1100 °C) ✓ for BNi-2/STEMET 1301 (1050–1070 °C) and the phosphorus grades (920–990 °C), ✗ for BNi-1 and BNi-5 (≥1145 °C)	✓ t_cr_ up to 70 μm (BNi-2, STEMET 1301)	✓ steels and Ni superalloys after the TLP hold (σjoint/σbase ≈ 1 for BNi-2 at the optimum clearance, Table 4); △ phosphorus grades on steels (0.4–0.5, Table 4)	✓ widths up to 200 mm [53,60]	✓
Fe–Cr–Si–B	✗ 1120–1220 °C for most steels (limit 1100 °C); applicable to steels with a higher limit (EP-823, Section 7.7)	△ amorphous–crystalline ribbon 40–45 μm [191]; t_cr_ of fully amorphous ribbon not reported	✓ steels (Fe–12Cr–Si–B on EP-823: 750 ± 90 MPa, base-metal level [191]); △ phosphorus grades (seam strength below the nickel counterparts [190])	✓ as cast [89,189,191]	✓ inexpensive
Ag–Cu–(Zn, Sn, In), rolled	✓ 600–900 °C	✗ low GFA; amorphization not required (ductile)	✓ universal family	✓ rolled foil and wire	△ Cd compositions excluded (REACH); silver is expensive
Al–Si (rods, cast ribbon)	✓ 577–615 °C	✗ no amorphous phase forms	✓ aluminum	△ semi-flexible cast ribbon	✓
Al–REM–TM (Al–Ni–Y, etc.)	✗ melt above the aluminum brazing window	△ glass former present, but no depressant	✗	✗ thin brittle ribbons	△ REM are expensive
Vitreloy-type BMGs (Zr–Cu–Al–Ni–Be)	✓ (T_liq_ 720 °C [54])	✓ record GFA	△ isolated demonstrations	✓ ribbon and bulk form	△ beryllium (special handling) and cost of one-off production
Pd–Ni–P, Pt–Cu–P	✓	✓ record GFA	✓ steels, heat-resistant alloys	✓	✗ cost of the noble metals

Legend: ✓, criterion met; △, met with reservations; ✗, not met, according to the thresholds of Table 2 (Section 2.2); “not assessed” marks the absence of the data required by Table 2. The entries refer to the representative grades named in Section 2.3 and Table 6. Entries that list several grades or base materials are mixed assessments: each symbol applies only to the item it precedes, and no single symbol is assigned to the family; for C1 the leading symbol is the rule-based grade of the whole brazing window of the family (a window that only partly lies at or below T_lim_ − 30 °C is graded with reservations, Table 2). Temperature limits: 1100 °C for most steels, ≈1250 °C for heat-resistant nickel alloys, 883 and 863 °C for unalloyed titanium and zirconium, ≈1000 °C for copper (Table 2). The bottom five rows correspond to the systems that did not pass the selection (negative control, see Section 2.3 and Section 4).

**Table 8:** Independence of the AWS A5.8 [15] classification and the ability to amorphize into ribbon.

	Amorphizes Into Ribbon	No Amorphous Ribbon Reported
AWS code exists	BNi-1, BNi-2, BNi-3, BNi-9, BNi-5a, BNi-6, BNi-7, BNi-12; BTi-1, BTi-2, BTi-3, BTi-5; BCo-1	BNi-5 (does not amorphize), BNi-10, BNi-11 (powder; amorphous ribbon not reported); BAg series Ag–Cu–Zn (rolled foil); BAlSi series Al–Si (rods)
No AWS code	Cu–Ni–Sn–P, Zr–Ti–Cu–Ni, Zr–Cu–Fe–Be, Ti–Zr–Be, Fe–Si–B	VPr24/32/36/37, G40NKh, G70NKh (powder and rolled stock); Ni–Si–Be (nanocrystalline ribbon)

## Data Availability

No new data were created or analyzed in this study. Data sharing is not applicable to this article.
